# Exploration of the Crystal Structure and Thermal and Spectroscopic Properties of Monoclinic Praseodymium Sulfate Pr_2_(SO_4_)_3_

**DOI:** 10.3390/molecules27133966

**Published:** 2022-06-21

**Authors:** Yuriy G. Denisenko, Victor V. Atuchin, Maxim S. Molokeev, Alexander E. Sedykh, Nikolay A. Khritokhin, Aleksandr S. Aleksandrovsky, Aleksandr S. Oreshonkov, Nikolai P. Shestakov, Sergey V. Adichtchev, Alexey M. Pugachev, Elena I. Sal’nikova, Oleg V. Andreev, Illaria A. Razumkova, Klaus Müller-Buschbaum

**Affiliations:** 1 Department of Inorganic and Physical Chemistry, Tyumen State University, 625003 Tyumen, Russia; yu.g.denisenko@gmail.com (Y.G.D.); kna@utmn.ru (N.A.K.); elenasalnikova213@gmail.com (E.I.S.); o.v.andreev@utmn.ru (O.V.A.); razumkova@list.ru (I.A.R.); 2 Department of General and Special Chemistry, Industrial University of Tyumen, 625000 Tyumen, Russia; 3 Institute of Inorganic and Analytical Chemistry, Justus-Liebig-University Giessen, 35392 Giessen, Germany; alexander.sedykh@anorg.chemie.uni-giessen.de (A.E.S.); klaus.mueller-buschbaum@anorg.chemie.uni-giessen.de (K.M.-B.); 4 Laboratory of Optical Materials and Structures, Institute of Semiconductor Physics, SB RAS, 630090 Novosibirsk, Russia; 5 Research and Development Department, Kemerovo State University, 650000 Kemerovo, Russia; 6 Department of Applied Physics, Novosibirsk State University, 630090 Novosibirsk, Russia; 7 Department of Industrial Machinery Design, Novosibirsk State Technical University, 630073 Novosibirsk, Russia; 8 R&D Center “Advanced Electronic Technologies”, Tomsk State University, Tomsk 634034, Russia; 9 Laboratory of Crystal Physics, Kirensky Institute of Physics, Federal Research Center KSC SB RAS, 660036 Krasnoyarsk, Russia; msmolokeev@mail.ru; 10 School of Engineering Physics and Radio Electronics, Siberian Federal University, 660041 Krasnoyarsk, Russia; 11 Department of Physics, Far Eastern State Transport University, 680021 Khabarovsk, Russia; 12 Laboratory of Coherent Optics, Kirensky Institute of Physics Federal Research Center KSC SB RAS, 660036 Krasnoyarsk, Russia; aleksandrovsky@kirensky.ru; 13 Institute of Nanotechnology, Spectroscopy and Quantum Chemistry, Siberian Federal University, 660041 Krasnoyarsk, Russia; 14 Laboratory of Molecular Spectroscopy, Kirensky Institute of Physics Federal Research Center KSC SB RAS, 660036 Krasnoyarsk, Russia; oreshonkov@iph.krasn.ru (A.S.O.); nico@iph.krasn.ru (N.P.S.); 15 School of Engineering and Construction, Siberian Federal University, 660041 Krasnoyarsk, Russia; 16 Institute of Automation and Electrometry, Russian Academy of Sciences, 630090 Novosibirsk, Russia; adish2@ngs.ru (S.V.A.); apg@iae.nsk.su (A.M.P.); 17 Research Department, Northern Trans-Ural Agricultural University, 625003 Tyumen, Russia; 18 Center for Materials Research (LaMa), Justus-Liebig-University Giessen, 35392 Giessen, Germany

**Keywords:** praseodymium sulfate, crystal structure, thermal analysis, thermal expansion anisotropy, photoluminescence, band structure, vibrational properties

## Abstract

Praseodymium sulfate was obtained by the precipitation method and the crystal structure was determined by Rietveld analysis. Pr_2_(SO_4_)_3_ is crystallized in the monoclinic structure, space group *C*2/*c*, with cell parameters *a* = 21.6052 (4), *b* = 6.7237 (1) and *c* = 6.9777 (1) Å, β = 107.9148 (7)°, *Z* = 4, *V* = 964.48 (3) Å^3^ (*T* = 150 °C). The thermal expansion of Pr_2_(SO_4_)_3_ is strongly anisotropic. As was obtained by XRD measurements, all cell parameters are increased on heating. However, due to a strong increase of the monoclinic angle β, there is a direction of negative thermal expansion. In the argon atmosphere, Pr_2_(SO_4_)_3_ is stable in the temperature range of *T* = 30–870 °C. The kinetics of the thermal decomposition process of praseodymium sulfate octahydrate Pr_2_(SO_4_)_3_·8H_2_O was studied as well. The vibrational properties of Pr_2_(SO_4_)_3_ were examined by Raman and Fourier-transform infrared absorption spectroscopy methods. The band gap structure of Pr_2_(SO_4_)_3_ was evaluated by ab initio calculations, and it was found that the valence band top is dominated by the p electrons of oxygen ions, while the conduction band bottom is formed by the d electrons of Pr^3+^ ions. The exact position of ZPL is determined via PL and PLE spectra at 77 K to be at 481 nm, and that enabled a correct assignment of luminescent bands. The maximum luminescent band in Pr_2_(SO_4_)_3_ belongs to the ^3^P_0_ → ^3^F_2_ transition at 640 nm.

## 1. Introduction

Rare earth (Ln) containing crystals exhibit exceptional material properties with wide-ranging technological significance [1,2,3,4]. The materials are widely used in solid-state laser devices, nonlinear optics and electronic and photonic systems because of their unique electron level configuration and specific chemical properties [5,6,7,8,9,10,11,12,13,14,15,16,17]. As to the crystal chemistry of Ln-containing compounds, it is based on the existence of the element range from La to Lu with a continuous variation of effective radius of Ln^3+^ ions that, in many cases, governs the boundaries of particular structure types [18]. Accordingly, structural, thermal and optical properties can be tuned by the substitution of Ln^3+^ ions. A lot of such inorganic crystal families can be found in the literature for different anion types, and the crystals with (SO_4_)^2−^ units are among less studied ones. As may be reasonably assumed, this state of things was formed due to the known effect of high hygroscopicity of sulfate compounds, and that greatly complicates their synthesis and use in precise electronic and optical technologies. Nevertheless, sulfate materials are traditionally applied in building industry, the extraction of Ln elements from natural and waste sources and catalysis [19,20,21,22,23,24,25,26,27].

In recent years, sulfate crystals have been actively studied in the general flow of searching new optical materials transparent in the UV spectral range, and many novel materials with interesting linear and nonlinear optical properties were discovered [28,29,30,31,32,33,34,35,36,37,38,39,40,41,42]. Some specific features were found in the coordination of (SO_4_)^2−^ anions in the crystal lattice [41]. However, there are no sufficient data on the structure of many known sulfate compounds for a proper classification and property analysis. In particular, despite the redundancy of the data on the crystal structures and properties of rare-earth sulfate hydrates Ln_2_(SO_4_)_3_·xH_2_O, most of the corresponding anhydrous phases with general composition Ln_2_(SO_4_)_3_ are not even structurally characterized. To date, only the crystal structures of two sulfates of light rare-earth elements, namely Nd_2_(SO_4_)_3_ [43] and Eu_2_(SO_4_)_3_ [44], have been described in detail. It was established that both phases crystallize in the monoclinic system, space group *C*2/*c*. As to sulfates of heavy rare-earth elements, crystal structures are available for Ln_2_(SO_4_)_3_, Ln = Y [45], Er [46] and Yb [47] compounds. These materials are predominantly crystallized in the orthorhombic system, space group *Pbcn*. Moreover, the noncentrosymmetric trigonal polymorphic modification was reported for Yb_2_(SO_4_)_3_ (space group *R*3*c*) [44], and a trigonal structure (space group *R*-3*c*) was observed in closely related sulfate Sc_2_(SO_4_)_3_ [48]. Thus, the crystal chemistry of Ln_2_(SO_4_)_3_ compounds is not simple, and the appearance of different structure types is possible depending on the Ln element and formation conditions.

The present study is aimed at the preparation of Pr_2_(SO_4_)_3_ and the evaluation of its structural, thermal and spectroscopic characteristics. This contribution allows evaluating the composition boundaries of the existence of monoclinic structure in anhydrous sulfates Ln_2_(SO_4_)_3_. As is known, praseodymium, due to its peculiar electronic structure, may be in different valence states and exhibits various coordination environments in the crystal lattice [49,50,51,52,53,54,55]. Praseodymium ions are able to accept an oxygen deficiency in oxide systems, thereby causing the photocatalytic activity of Pr-containing compounds [55,56,57,58,59,60,61]. The systems with the Pr^3+^ ions could exhibit interesting spectroscopic properties as promising optical and luminescent materials [62,63,64,65,66,67,68]. Accordingly, the characterization of Pr-containing sulfates is of particular interest. In this work, anhydrous sulfate Pr_2_(SO_4_)_3_ was synthesized by the chemical precipitation method, and its structural and thermophysical parameters were determined on the base of X-ray diffraction measurements. The thermal stability of the sulfate was evaluated by simultaneous DTA/TG measurements. The vibrational properties of Pr_2_(SO_4_)_3_ were obtained by IR and Raman spectral analyses. Then, photoluminescence effects were comparatively evaluated at 77 and 300 K.

## 2. Methods and Materials

Praseodymium (III) sulfate Pr_2_(SO_4_)_3_ was synthesized by the precipitation from a solution of Pr(NO_3_)_3_. Pr_6_O_11_ (99.99%, ultrapure, TDM-96 Ltd., Ekaterinburg, Russia), concentrated nitric acid solution (C(HNO_3_) = 14.6 mol/L, ultrapure, Vekton Ltd., St. Petersburg, Russia) and concentrated sulfuric acid solution (C(H_2_SO_4_) = 17.9 mol/L, ultrapure, Vekton Ltd., St. Petersburg, Russia) were used as the starting reagents. Weighing the dry reagents was carried out on an analytical balance of the accuracy of 0.1 mg. Praseodymium oxide, prior to weighing, was calcined in a muffle furnace at the temperature of 1000 °C for 12 h to remove the gases adsorbed from the air and the products of their interaction with the Pr_6_O_11_ surface. The acid solutions were measured by means of glass measuring cylinders with an accuracy of 0.1 mL.

First, the 2.9866 g Pr_6_O_11_ charge was placed in a 100 mL glass round-bottomed flask. Then, 3.6 mL of the concentrated nitric acid solution was added in small portions. The reaction mixture was heated with a continuous stirring until the oxide was completely dissolved. As a result, the praseodymium (III) nitrate solution was obtained by redox reaction:

Pr_6_O_11_ + 18HNO_3_ → 6Pr(NO_3_)_3_ + 9H_2_O + O_2_ (1)

After cooling the solution to room temperature, 1.6 mL (an excess of 10%) of the concentrated sulfuric acid solution was added to the flask in small portions, not allowing a strong reheating of the reaction mixture. The reaction results in the praseodymium sulfate precipitation:

2Pr(NO_3_)_3_ + 3H_2_SO_4_ → Pr_2_(SO_4_)_3_↓ + 6HNO_3_ (2)

After the precipitation, the mixture was distilled to a dry residue. The praseodymium sulfate powder was additionally calcined in a tubular furnace at 500 °C to remove the adsorbed acid and then annealed in a muffle furnace at the same temperature for 7 days to form the final powder product. According to the synthesis steps described above, 4.9672 g of praseodymium sulfate powder were obtained. The yield of the target product is 99% of the theoretical level. According to the gravimetric analysis, the content of sulfate ions in the resulting compound is 50.58%. At the theoretical value of 50.56% for Pr_2_(SO_4_)_3_, the possible determination error is 0.5%, which corresponds to the relative error for this analytical method. As seen in the photo shown in Figure S1a, the synthesized powder of praseodymium sulfate has a light green tint, which is a common characteristic of Pr^3+^-containing oxides.

Praseodymium (III) sulfate octahydrate Pr_2_(SO_4_)_3_·8H_2_O was obtained by the crystallization from an aqueous saturated solution at room temperature in a vacuum desiccator under reduced pressure. A saturated solution was prepared by dissolving anhydrous praseodymium (III) sulfate Pr_2_(SO_4_)_3_ (chemically pure) weighing 2.50 g in 100 mL of deionized water at the temperature of 20 °C. The precipitate formed by crystallization was separated from the mother liquor, squeezed between filter paper sheets and kept at room temperature on watching glass in a desiccator with calcined silica gel to reach a constant weight. Thus, light green shiny crystals of praseodymium (III) sulfate octahydrate Pr_2_(SO_4_)_3_·8H_2_O were obtained. A photo of this powder product is shown in Figure S1b. As is evident, the colors of both Pr_2_(SO_4_)_3_ and Pr_2_(SO_4_)_3_·8H_2_O are in the green color spectrum, but the tints are different, which could be attributed to the difference in the crystal structure and the presence of H_2_O units.

The structural properties of the powder samples were obtained by the X-ray diffraction analysis with the use of a Bruker D8 ADVANCE powder diffractometer (Cu-Kα radiation) and linear VANTEC detector. The step size of 2θ was 0.016°, and the integration time was 3 s per step. First, to evaluate the chemical stability of the Pr_2_(SO_4_)_3_ sample, several XRD patterns were collected each 30 min in contact with the laboratory air at room temperature, normal pressure and humidity (Figure S2). As the X-ray patterns noticeably changed with the exposure time increase, it was concluded that the sample absorbs water from the air, leading to the formation of intermediate hydrated phases. Therefore, to exclude the hydration effects, the powder data for Rietveld analysis were collected at 150 °C using an Anton Parr thermal attachment. Fitting of the profile, searching the crystal structure and Rietveld refinements were performed by using TOPAS 4.2 [69]. In the determination of thermophysical parameters, the XRD patterns were recorded using the same Bruker D8 ADVANCE powder diffractometer (Cu-Kα radiation) and linear VANTEC detector. The Anton Parr thermal attachment was applied for the temperature control. Nine XRD patterns were measured in the temperature range of 30–270 °C with the 30 °C step and 0.4 s exposition time to obtain the thermal dependences of cell parameters.

All first principal calculations were performed using the density functional theory approach, as implemented in the CASTEP code [70]. The 4f^3^ 5s^2^ 5p^6^ 6s^2^, 3s^2^3p^4^ and 2s^2^2p^4^ valence electron configurations were considered for Pr, S and O atoms, respectively. The local density approximation plus U (LDA + U) based on the Perdew and Zunger parametrization [71] of the numerical results of Ceperley and Alder [72] was used for the calculation. The Hubbard U energy term for the Pr 4f orbital was taken as U_f_ = 6 eV. The C19 on-the-fly-generated ultrasoft pseudopotentials were used, and the cutoff energy for the plane basis was chosen as that equal to 630 eV. The tolerance level for the geometry optimization was chosen as 5.0 × 10^−4^ eV/Å for the maximal force and 0.02 GPa for the maximal stress. The Monkhorst-Pack *k*-point integration network of the Brillouin zone was taken as 3 × 3 × 3.

The particle morphology was observed by Scanning Electron Microscopy (SEM) with the use of an electron microscope JEOL JSM-6510LV. An X-ray energy-dispersive analyzer Oxford Instruments X-Max 20 mm^2^ was applied to determine the constituent element ratio. The chemical composition measurements were carried out with the use of a pressed tablet. The accuracy in the element content determination was equal to ±0.2%.

The thermal analysis in an argon flow was carried out by a Simultaneous Thermal Analysis (DTA/TG) equipment 499 F5 Jupiter NETZSCH (Germany). The powder sample was inserted into an alumina crucible. The heating rate was 3 °C/min. For the enthalpy determination, the equipment was calibrated with the use of standard substances, such as In, Sn, Bi, Zn, Al, Ag, Au and Ni. The heat effect characteristics were determined with the package Proteus 6 [73]. The peak temperatures and areas in parallel experiments were reproduced with an inaccuracy lower than 3%. The kinetic parameters determination was based on Kissinger formula [74] in the linearized form:

$$\frac{1}{T}=\frac{1}{E}\cdot R\ln\frac{b}{T^{2}}-\frac{R}{E}\ln\frac{AR}{E}$$

where *T* is the temperature with the maximum reaction rate; *b* is the heating rate, dps; *E* is the activation energy; *A* is the pre-exponential factor. The examples of the practical application of this formula to the analysis of topochemical reactions in different complex systems can be found elsewhere [35,75,76,77].

The infrared (IR) absorption spectrum was recorded with a Fourier-transform spectrometer VERTEX 70 V (Bruker, Billerica, MA, USA) in the spectral range from 400 to 1600 cm^−1^ with the spectral resolution of 4 cm^−1^. The spectrum was recorded for a tablet sample shaped as about 0.4 mm thick tablet of 13 mm in diameter and the weight of 0.1203 g. The tablet was prepared as follows: 0.0030 g of Pr_2_(SO_4_)_3_ was thoroughly ground with 0.12 g of KBr. The Globar was used as a light source, and it was equipped with a KBr wide-range beamsplitter (Vilnius, Lithuania) and RT-DLaTGS as a detector.

The Raman experiment with the excitation by a Nd:YAG laser (1064 nm) was carried out on an IR Raman spectrometer (Bruker Optik GmbH), which consists of a Vertex 85 IR spectrometer and a Ram II Raman attachment. The laser output radiation power was as high as 100 mW, and the spectral resolution of the spectrometer was equal to 4 cm^−1^. The Raman measurements with the excitation at 532.1 nm were performed using a Millennia solid-state laser (Spectra Physics, Milpitas, CA, USA) and a Trivista 777 triple-grating spectrometer (Princeton Instruments, Trenton, NJ, USA). The Raman spectrum of the Pr_2_(SO_4_)_3_ powder was recorded at ambient temperature in the backscattering geometry in the frequency range from 30 to 1400 cm^−1^ without choosing the polarization. The spectral resolution was as high as ~1 cm^−1^.

For measuring photoluminescence properties, solid samples were filled in spectroscopically pure quartz glass cuvettes and examined either at room temperature or at 77 K (for the latter using a special liquid nitrogen-filled Dewar assembly FL-1013, HORIBA, Singapore). The excitation and emission spectra were recorded with a HORIBA Jobin Yvon Spex Fluorolog 3 spectrometer equipped with a 450 W Xe short-arc lamp, double-grated excitation and emission monochromators, and a photomultiplier tube (R928P) using the FluoroEssence™ software. Both excitation and emission spectra were corrected for the spectral response of the monochromators and the detector using the correction files provided by the manufacturer. The excitation spectra were additionally corrected for the spectral distribution of the lamp intensity by the use of a photodiode reference detector.

## 3. Results and Discussion

### 3.1. Structural Properties

**Figure 1:**
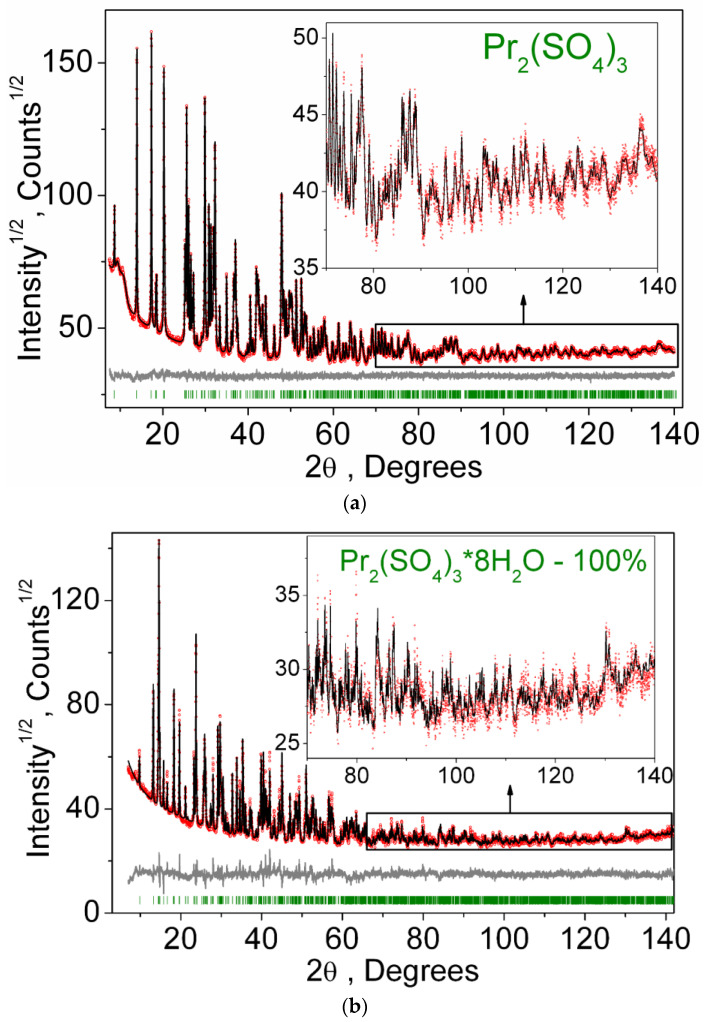
XRD patterns and difference Rietveld plots of (**a**) Pr_2_(SO_4_)_3_, as obtained at 150 °C, and (**b**) Pr_2_(SO_4_)_3_·8H_2_O.

**Figure 2:**
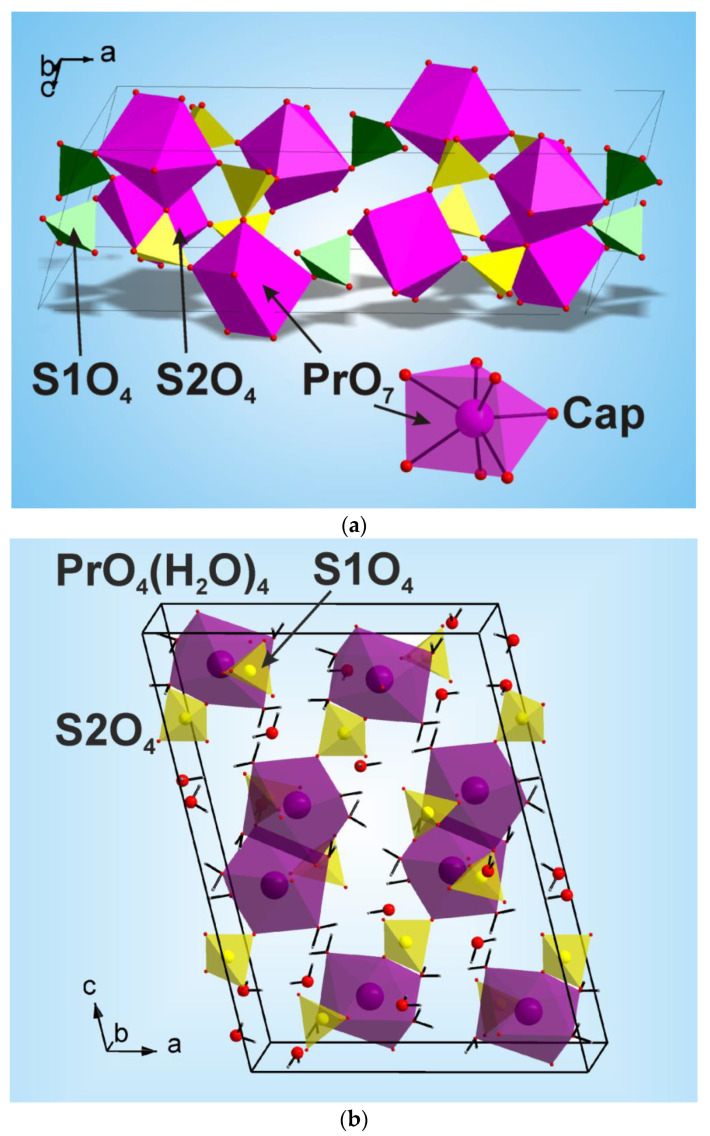
Crystal structures of (**a**) Pr_2_(SO_4_)_3_ and (**b**) Pr_2_(SO_4_)_3_·8H_2_O. The unit cell is outlined. Lone atoms are omitted for clarity.

The XRD pattern recorded for the Pr_2_(SO_4_)_3_ sample is shown in Figure 1a. All reflections were successfully indexed by the C-centered monoclinic cell (*a* = 21.586, *b* = 6.715 and *c* = 6.969 Å, β = 107.93°, GoF = 53.7), and the analysis of reflection extinction showed that the most probable space groups are *C*2/*c* or *Cc*. It should be noted that, earlier, the Pr_2_(SO_4_)_3_ structure was indexed by the monoclinic unit cell, but with twice bigger asymmetric unit cell volume (*a* = 21.71, *b* = 6.941 and *c* = 6.722 Å, β = 109.03°, space group *P*2/*a*) [78]. As far as our unit cell has a higher symmetry and a lower cell volume of asymmetric part, it was chosen for the structure analysis. Moreover, from two possible space groups *C*2/*c* and *Cc*, the former was chosen as a starting point. The crystal structure was solved using a simulated annealing procedure applied to the randomized coordinates of one Pr^3+^ ion and two (SO_4_)^2−^ tetrahedra [79]. The dynamic occupancy correction of the atoms was used to merge the ions falling in special positions [79,80]. After the calculations, a solution was found with small *R*-factors. The crystal structure contains one Pr^3+^ ion in general position (8f), one (SO_4_)^2−^ tetrahedron in special site (4e) and one (SO_4_)^2−^ tetrahedron in general site (8f), as shown in Figure 2a. The refinement in this model was stable and given the low *R*-factors, as presented in Table 1 and Figure 1a. The atom coordinates and main bond lengths are given in Tables S1 and S2, respectively. The structural analysis of Pr_2_(SO_4_)_3_ with the use of program PLATON [81] does not reveal any additional elements of symmetry, and it proves the selection of space group *C*2/*c*.

The bond valence sum calculated for the Pr^3+^ ion using values *r*_0_ = 2.138 Å and *b*_0_ = 0.37 [82] and taking into account short bond lengths d(Pr–O) in the range of 2.349(5)–2.530(7) Å without long bond lengths (2.716(7)–2.792(8) Å) gave the value BVS(Pr^3+^) = 3.11, which is close to the formal valence state 3+ of the Pr ion. Similar calculations for all S^6+^ ions were made using *r*_0_ = 1.624 Å, *b*_0_ = 0.37 [75] yield BVS(S1) = 5.71 and BVS(S2) = 6.52, which are also in a good agreement (less than ±10% of average value) with the formal valence state 6+ of S ions. Thus, accounting for short bond lengths d(Pr–O), one can assume the existence of monocaped trigonal PrO_7_ prisms in the structure (Figure 2a). These prisms are joined with SO_4_^2−^ tetrahedra by nodes forming a 3D net. The topological analysis of the net, using the simplification that S1O_4_, S2O_4_ and PrO_7_ are just nodes, reveals that this is a three-nodal (4-c)(5-c)_2_(9-c)_2_ net with the point symbol (3^2^.4^2^.5^2^)(3^2^.4^7^.5)_2_(3^6^.4^14^.5^8^.6^8^)_2_, which is new [83]. Thus, presently, this family of monoclinic anhydrous sulfates includes three compounds Ln_2_(SO_4_)_3_ (Ln = Pr, Nd, Eu), for which structural parameters are known [42,43]. However, with a high probability, it can be assumed that Ln_2_(SO_4_)_3_ (Ln = Pm, Sm) have structures of the same type.

**Figure 3:**
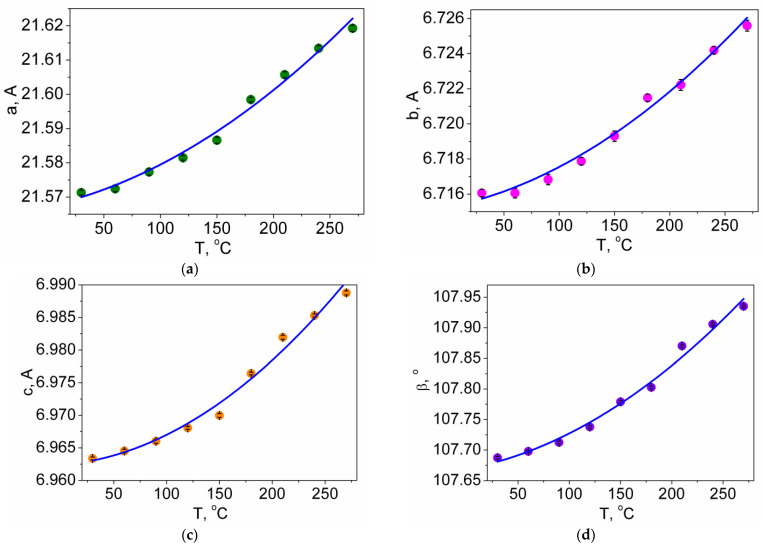

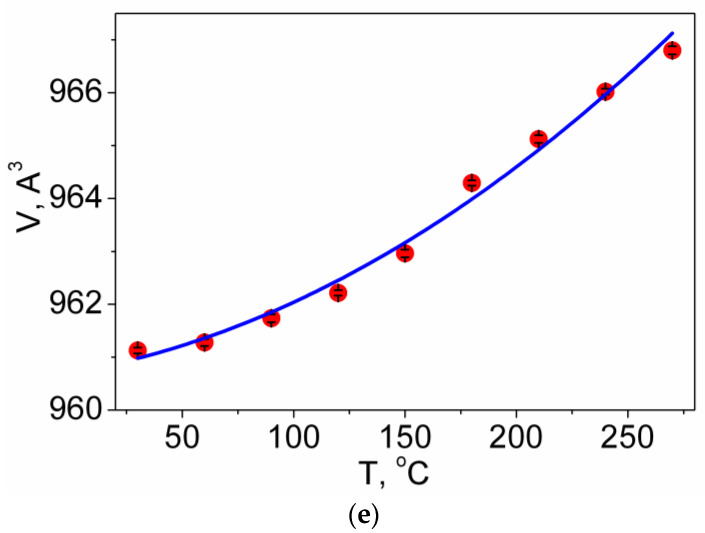
Thermal dependence of Pr_2_(SO_4_)_3_ cell parameters: (**a**) *a*(T); (**b**) *b*(T); (**c**) *c*(T); (**d**) β(T); (**e**) *V*(T).

**Figure 4:**
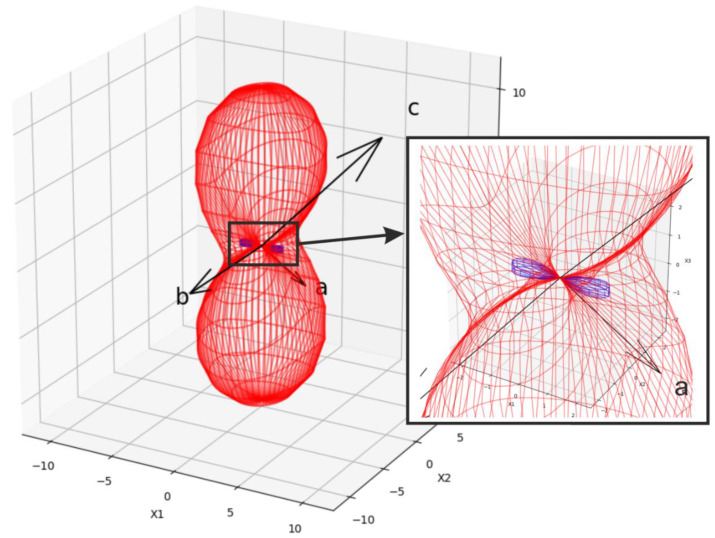
Thermal expansion tensor of Pr_2_(SO_4_)_3_ calculated for the temperature range of 30–150 °C. Contraction is shown in blue color.

As seen in Figure 3, heating the Pr_2_(SO_4_)_3_ sample from 30 to 270 °C leads to an increase of all cell parameters (Table S3) with δ*a*~0.23%, δ*b*~0.15% and δ*c*~0.39%, showing the 3D net expansion in all crystallographic directions accompanied by an increase of the monoclinic angle β. The continuous variation of the cell parameters (Figure 3) and freedom from the reflection splitting and/or superstructure reflections in the powder patterns (Figure S3) indicate the absence of structural phase transitions in the range of 30–270 °C. Therefore, we can suggest that Pr_2_(SO_4_)_3_ at room temperature also adapts the *C*2/*c* space group. The thermal expansion tensor of Pr_2_(SO_4_)_3_ is shown in Figure 4. As is evident, the crystal expansion is strongly anisotropic. Moreover, there is a direction along which a contraction appears on heating, mainly due to a monoclinic angle increase.

The XRD pattern recorded for the Pr_2_(SO_4_)_3_·8H_2_O sample is shown in Figure 1b. All peaks of the pattern were indexed according to the known structure of Pr_2_(SO_4_)_3_·8H_2_O [23], and, therefore, this structure was used as the initial model. The refinements were stable and gave low R-factors, as listed in Table 2 and shown in Figure 1b. The atom coordinates and main bond lengths are in Tables S4 and S5, respectively. Hydrogen atoms were placed in ideal sites and their coordinates were fixed during a further crystal structure refinement. The asymmetric part of the unit cell contains one Pr ion, two S ions, six O ions and four H_2_O molecules. The Pr^3+^ ion is coordinated by four O ions and four H_2_O molecules forming a PrO_4_(H_2_O)_4_ antisquare prism. Each S ion is coordinated by four O ions forming a SO_4_ tetrahedra. The SO_4_ tetrahedra are linked with PrO_4_(H_2_O)_4_ polyhedra by edges and nodes forming a 3D net, as displayed in Figure 2b.

The crystallographic data of the crystal structures of Pr_2_(SO_4_)_3_ and Pr_2_(SO_4_)_3_·8H_2_O are deposited in Cambridge Crystallographic Data Centre (CSD #2167673-2167674). The data can be down loaded from the site (www.ccdc.cam.ac.uk/data request/cif, accessed on 20 April 2022).

### 3.2. Electronic Properties

**Figure 5:**
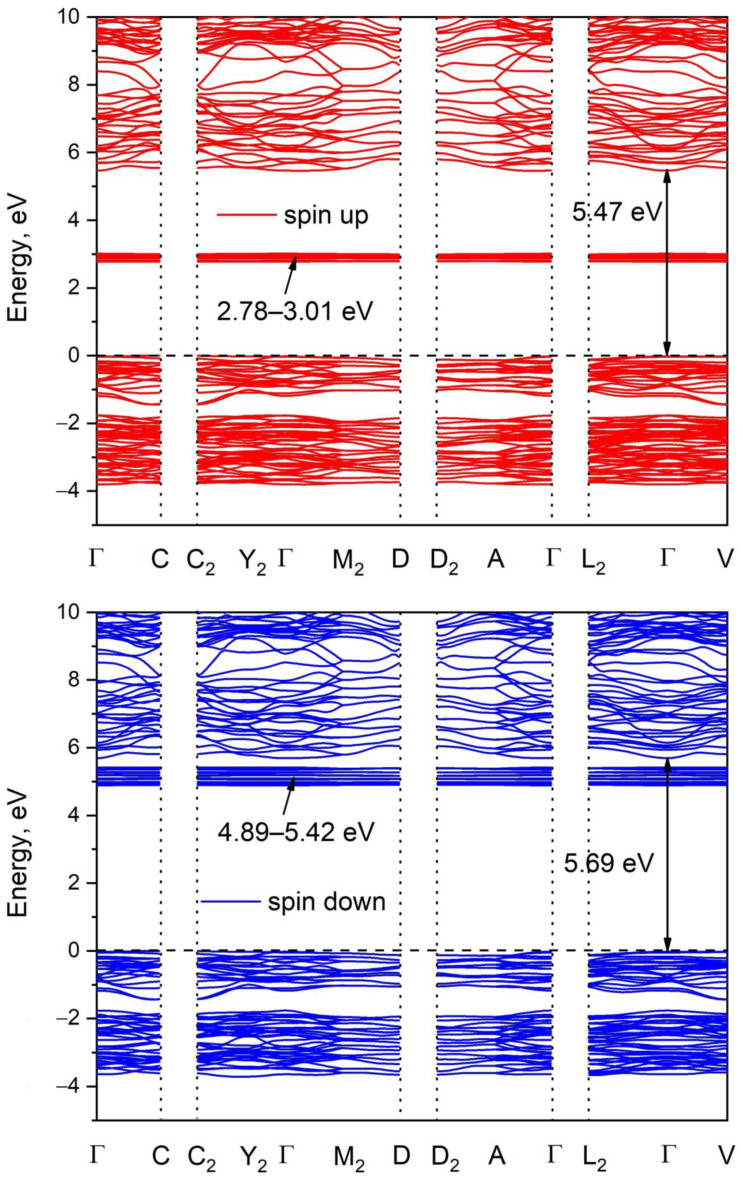
Calculated band structure of Pr_2_(SO_4_)_3_. The lower panel is for spin up and the upper panel is for spin down.

**Figure 6:**
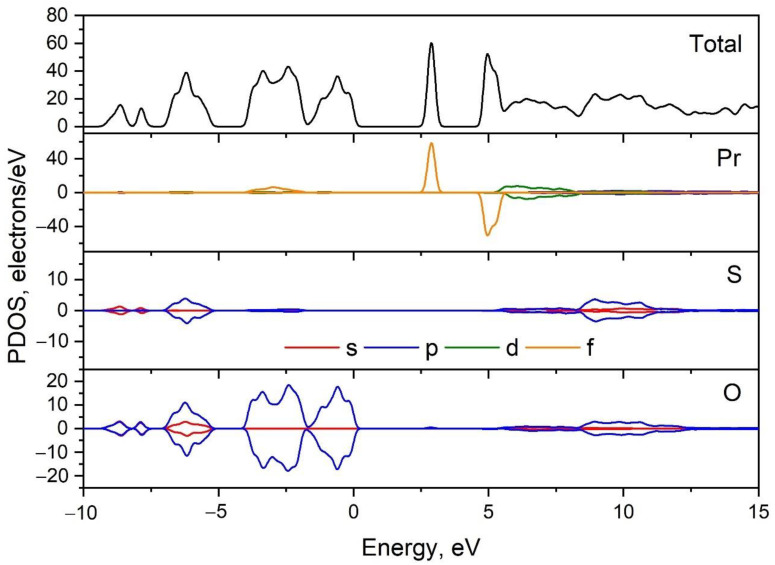
Calculated partial density of states in Pr_2_(SO_4_)_3_.

The Brillouin zone (BZ) image and the calculated electronic band structure of Pr_2_(SO_4_)_3_ are shown in Figure 5 and Figure S4, respectively. The paths along high symmetry points of the BZ are selected as follows: Γ–C, C_2_–Y_2_–Γ–M_2_–D, D_2_–A–Γ, L_2_–Γ–V and the coordinates of these points are: Γ(0,0,0), C(−0.277, 0.277,0), C_2_(−0.723, −0.277, 0), Y_2_(−0.5, −0.5, 0), M_2_(−0.5, −0.5, 0.5), D(−0.749, −0.251, 0.5), D_2_(−0.251, 0.251, 0.5), A(0, 0, 0.5), L_2_(−0.5, 0, 0.5), V_2_(−0.5, 0, 0). As praseodymium is related to lanthanides, the spin up and spin down band structures were calculated. According to the results shown in Figure 5, Pr_2_(SO_4_)_3_ is a direct band gap compound. The valence band maximum (VBM) and conduction band minimum (CBM) are located in the center of BZ. The calculated spin up band gap is equal to 5.47 eV, while the spin down band gap is as high as 5.69 eV. It should be noted that flat narrow electronic branches are observed at 2.78–3.01 eV in a spin up band structure and at 4.89–5.42 eV in a spin down band structure. To understand the nature of these branches and the nature of band gap, the partial density of electronic states is presented in Figure 6. From the curve observation, it can be concluded that the flat branches pointed above are formed by the f electronic states of Pr. The valence band top is dominated by the p electrons of oxygen ions, while the conduction band bottom is formed by the d electrons of Pr^3+^ ions.

### 3.3. Vibrational Properties

**Figure 7:**
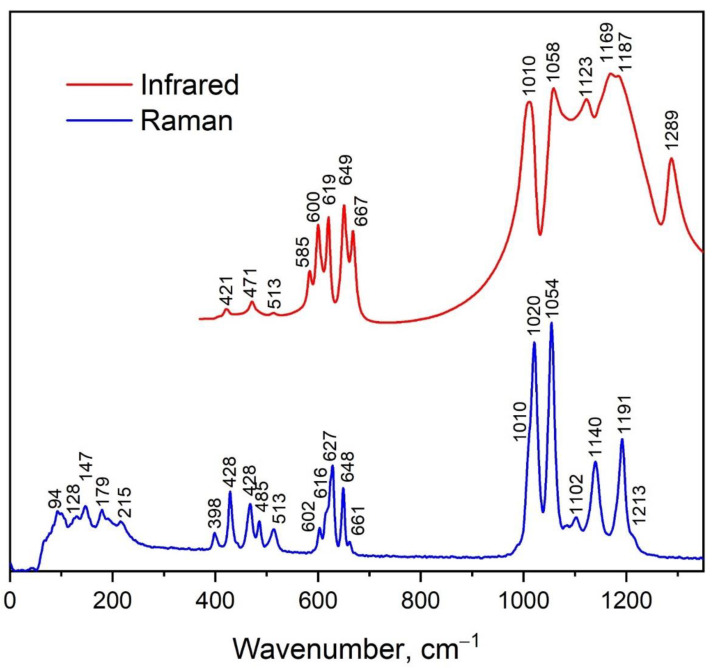
Raman and infrared spectra of Pr_2_(SO_4_)_3_.

There are 34 atoms in the primitive cell of Pr_2_(SO_4_)_3_ and the symmetry analysis leads to the following distribution of the 102 phonon modes between the irreducible representations at the center of Brillouin zone: Γ_vibr_ = 25*A_g_* + 25*A_u_* + 26*B_g_* + 26*B_u_* where acoustic modes are Γ_ac_o_ustic_ = *A_u_* + 2*B_u_*, and the remaining modes are optical. The *g*-labeled modes are Raman active, while the *u*-labeled modes are infrared active [84]. The vibrational spectra obtained for powder Pr_2_(SO_4_)_3_ are presented in Figure 7. The comparison of the Raman spectra recorded with the use of 1064 and 532.1 nm laser wavelengths is shown in Figure S5 and excellent relation of the spectra is evident. Thus, the luminescence lines do not appear under the excitation at 1064 and 532.1 nm and both wavelengths can be used for precise measurements of the Raman spectra of Pr^3+^-containing crystals. In the Pr_2_(SO_4_)_3_ structure, the SO_4_ tetrahedra occupy two crystallographically independent positions, namely, *C*_1_ and *C*_2_. As is known, free [SO_4_]^2−^ units have the *T_d_* symmetry, and the characteristic wavenumbers of normal vibrations of this ion group were listed in [85]. The correlation between internal vibrations of the free SO_4_ tetrahedra with the *T*_d_ symmetry, sites symmetry and factor group symmetry of the unit cell is shown in Table 3. Herein, the mode *ν*_1_ (*A*_1_) is symmetric stretching vibration, *ν*_3_ (*F*_2_) is antisymmetric stretching vibration and *ν*_2_ (*E*) and *ν*_4_ (*F*_2_) are symmetric and antisymmetric bending vibrations. The shapes of the vibrational spectra of Pr_2_(SO_4_)_3_ and Eu_2_(SO_4_)_3_ [43] powders are quite similar. This can be explained by the fact that the structures of these compounds are described in the same space groups and have the same number of SO_4_ tetrahedra in the same positions. However, due the differences in [SO_4_]^2−^ bond lengths, there is a slight shift in the spectral peaks, which is especially clear in the range of ν_1_ vibrations, as shown in Figure S6.

According to Table 3, the high wavenumber part (above 950 cm^−1^) of Raman and infrared spectra of Pr_2_(SO_4_)_3_ powder is correspondent to the stretching vibrations of SO_4_^2−^ ions. The spectral bands related to each symmetric stretching vibration of SO_4_ are clearly seen in the Raman spectrum at 1010, 1020 and 1054 cm^−1^, as seen in Figure 7 and Figure S7. The remaining Raman bands in this region are attributed to antisymmetric stretching vibrations. The broad band observed at 1010 cm^−1^ in the infrared spectrum should consist of three overlapped bands corresponding to *ν*_1_ vibrational modes, and the bands above 1030 cm^−1^ are related to antisymmetric stretching vibrations. The *ν*_4_ vibrations are located in the range of 595–670 and 575–675 cm^−1^ in Raman and infrared spectra, respectively (Figure 7 and Figure S8). The *ν*_2_ modes are observed in the Raman spectrum between 380 and 520 cm^−1^. Other Raman bands revealed below 250 cm^−1^ attributed to the rotation of SO_4_^2−^ and translational vibrations of the structural units. Thus, we can say that positions of spectral bands and their number are in agreement with group-theoretical analysis data for the Pr_2_(SO_4_)_3_ XRD-solved structure.

**Figure 8:**
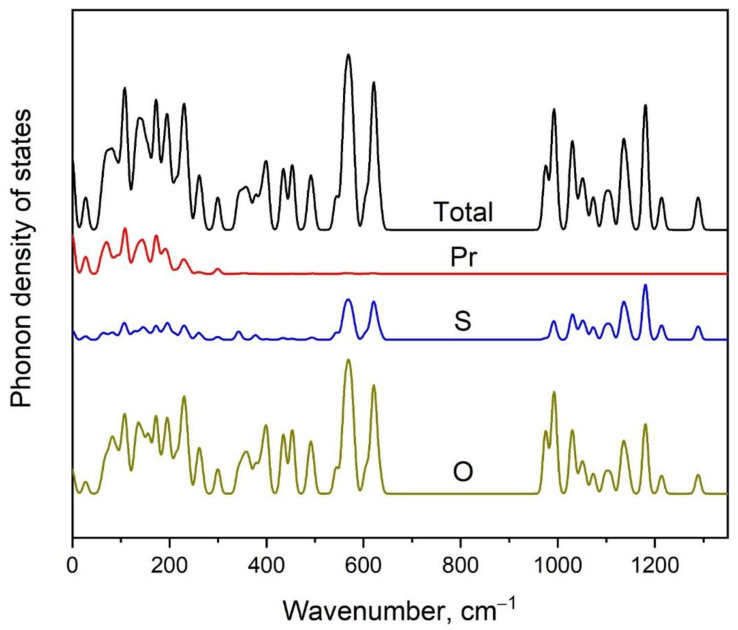
Calculated phonon density of states in Pr_2_(SO_4_)_3_.

The calculated partial phonon density of states is shown in Figure 8 and the presented data can be summarized as follows: the vibrations of SO_4_ tetrahedra dominated in the Raman and infrared spectra at wavenumbers above 250 cm^−1^, while the low wavenumber region is characterized by vibrations of all kinds of ions.

### 3.4. Thermal Properties

**Figure 9:**
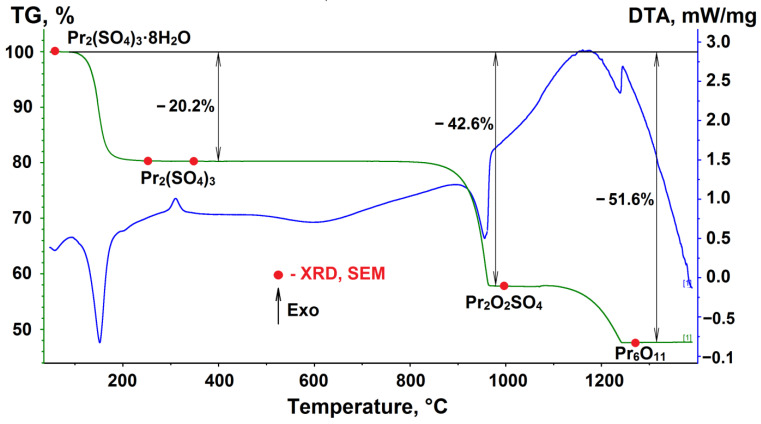
Simultaneous DTA/TG of Pr_2_(SO_4_)_3_.

The known problem with sulfates is their increased hygroscopicity. Upon obtaining functional materials based on lanthanide sulfates, important issues are the processes occurring during the dehydration of the corresponding salts. Pyrohydrolysis, often proceeding during the dehydration of salts, can significantly affect the properties of sulfate materials. In this relation, the TG/DTA data of praseodymium sulfate octahydrate were recorded on heating in the temperature range of 25–1400 °C in the argon atmosphere, as shown in Figure 9. According to the TG data in the temperature range of 73–210 °C, the mass loss is 20.2%, which allows us to draw up the process equation:

Pr_2_(SO_4_)_3_ × 8H_2_O → Pr_2_(SO_4_)_3_ + 8H_2_O (3)

**Figure 10:**
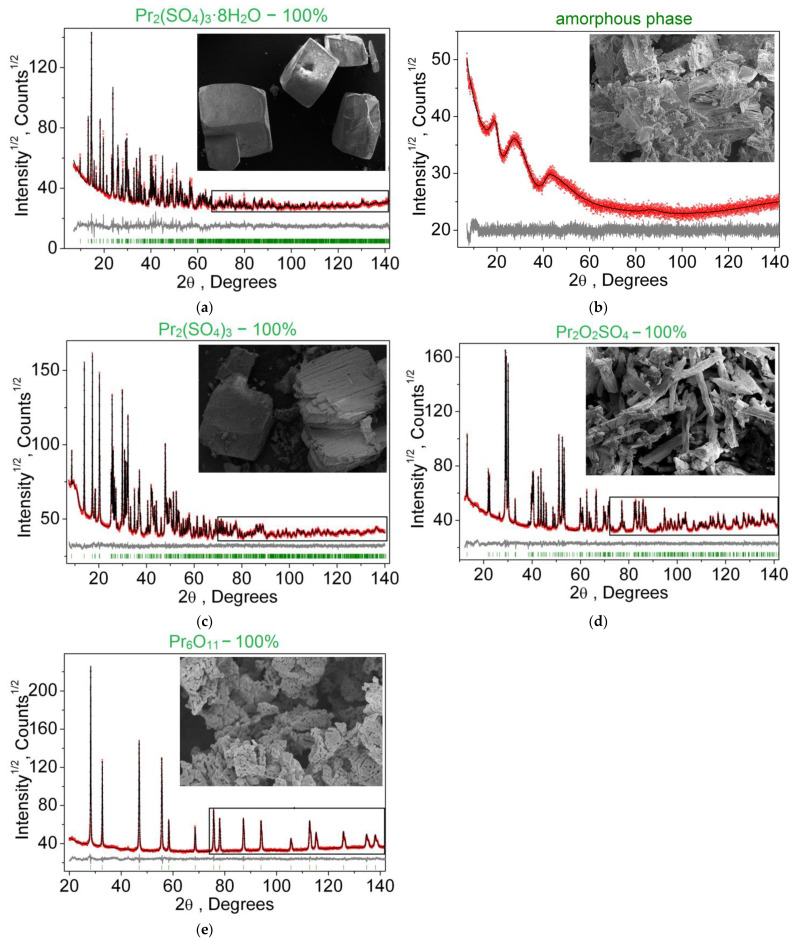
Difference Rietveld plots and microstructure transformation of polycrystalline samples Pr_2_(SO_4_)_3_·8H_2_O subjected to heat treatment at temperatures: (**a**) 25 °C (room temperature); (**b**) 250 °C; (**c**) 350 °C; (**d**) 1000 °C; (**e**) 1270 °C.

The dehydration proceeds in one stage despite the crystallo-chemical inequality of water molecules entering the structure [86]. In the interval of 350–370 °C, in all recorded DTA curves, a low-intensity peak of heat release was detected. To identify the source of this effect, isothermal treatments of praseodymium sulfate octahydrate were carried out at 250 °C and 350 °C. In both cases, the mass loss corresponds to the full dehydration of the samples. According to the X-ray phase analysis and electron microscopy, the starting octahydrate is represented by highly faceted crystals ranging in size from 5 to 20 μm (Figure 10a). Heating the Pr_2_(SO_4_)_3_ × 8H_2_O sample to 250 °C results in the formation of an X-ray amorphous product obtained by the dehydration process (Figure 10b). Obviously, the water vapor moving to the surface results in the particle destruction. The sample heating to 350 °C results in a polycrystalline powder of anhydrous Pr_2_(SO_4_)_3_ (Figure 10c), which was obviously formed via the recrystallization of the amorphous powder obtained at the initial stage of dehydration. Therefore, the presence of the heat release peak on the DTA curve is caused by the crystallization of the amorphous phase of Pr_2_(SO_4_)_3_.

Further decomposition of Pr_2_(SO_4_)_3_ on heating occurs in two steps. In the first step, in the temperature range of 850–970 °C, two sulfate groups undergo decomposition, resulting in the formation of praseodymium oxysulfate (Figure 10d):

Pr_2_(SO_4_)_3_ → Pr_2_O_2_SO_4_ + 2SO_2_ + O_2_ (4)

In the second step, in the temperature range 1100–1250 °C, the remaining sulfate groups were decomposed. According to the X-ray phase analysis, a mixed praseodymium oxide Pr_6_O_11_ is formed (Figure 10e) as the final product of the reaction:

6Pr_2_O_2_SO_4_ → 2Pr_6_O_11_ + 6SO_2_ + O_2_ (5)

The formation of intermediate oxide Pr_6_O_11_ is characteristic of the decomposition of oxygen-containing praseodymium compounds, just as the formation of CeO_2_ is typical of the corresponding cerium compounds [87] and Tb_4_O_7_ for terbium [88]. 4*f*-electron shell structures enhance the effect on the thermodynamic characteristics of compounds while simplifying the chemical composition.

On the base of reliable data on the phase composition of the compounds formed by thermal transformations, as well as the established values of the enthalpies of these transformations, we can write the thermochemical equations of the processes related to the Pr_2_(SO_4_)_3_·8H_2_O decomposition on heating:

Pr_2_(SO_4_)_3_·8H_2_O (monocl) → Pr_2_(SO_4_)_3_ (monocl) + 8H_2_O (gas); ∆H° = 108.9 kJ/mol (6)

Pr_2_(SO_4_)_3_ (monocl) → Pr_2_O_2_SO_4_ (monocl) + 2SO_2_(gas) + O_2_ (gas); ∆H° = 499.8 kJ/mol (7)

6Pr_2_O_2_SO_4_ (monocl) → 2Pr_6_O_11_ (cub) + 6SO_2_(gas) + O_2_ (gas); ∆H° = 245.5 kJ/mol (8)

Using the data on the formation enthalpies of binary compounds Pr_6_O_11_ [89], SO_2_ [90] and H_2_O [91], the enthalpies of praseodymium sulfates formation (Table 4) were successively calculated: Pr_2_O_2_SO_4_ (according to reaction (8)), Pr_2_(SO_4_)_3_ (according to reaction (7)) and Pr_2_(SO_4_)_3_·8H_2_O (according to reaction (6)).

To study the kinetics of the thermal decomposition processes of Pr_2_(SO_4_)_3_·8H_2_O, the sample thermal analysis was carried out at different heating rates: 3, 5, 10 and 15 °C/min. Based on the DTA data obtained at different heating rates (Figures S9 and S10), the kinetic parameters of the processes were calculated (Table 5). As can be seen, the increase in the activation energy during the transition from the dehydration process to the processes of sulfate decomposition is somewhat compensated by the increase in the pre-exponential factor value, which actually reflects the increase in the favorable steric factor. In general, in accordance with a significant expansion of the peaks in the DTA curves and, accordingly, with a significant increase in the activation energy of high-temperature processes for the decomposition of sulfate Pr_2_(SO_4_)_3_ and oxysulfate Pr_2_O_2_SO_4_, we can note their significant kinetic complexity, compared with the dehydration of crystalline hydrate Pr_2_(SO_4_)_3_·8H_2_O.

A comparison of the thermal decomposition of praseodymium sulfate octahydrate with the corresponding crystalline hydrate of europium sulfate Pr_2_(SO_4_)_3_·8H_2_O indicates a greater kinetic stability of Pr_2_(SO_4_)_3_·8H_2_O and Pr_2_(SO_4_)_3_, compared with the corresponding europium compounds, and a reduced stability of Pr_2_O_2_SO_4_ compared to Eu_2_O_2_SO_4_ [92]. This fact has, obviously, energetic reasons and is in a good agreement with the enthalpies of compound formation.

### 3.5. Luminescent Properties

**Figure 11:**
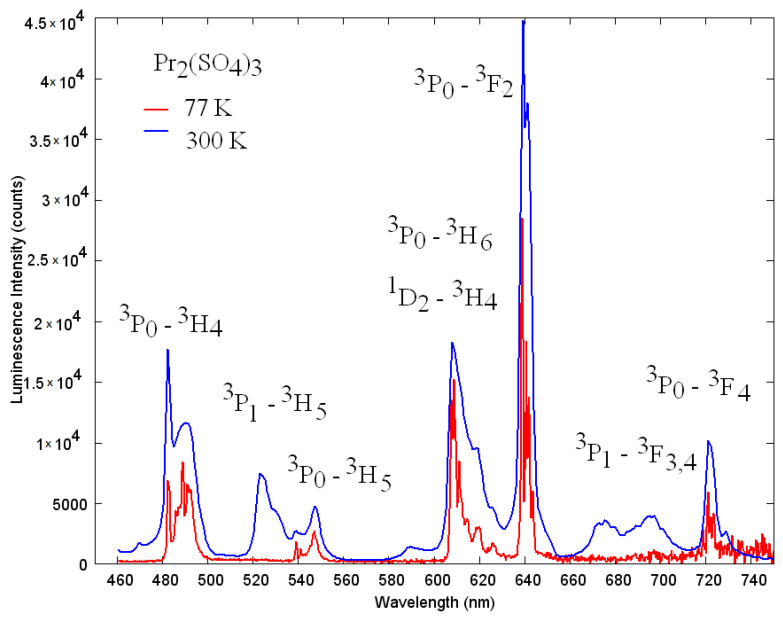
High resolution emission spectra of Pr_2_(SO_4_)_3_ at room temperature (blue) and at 77 K (red) excited at 440 nm.

**Figure 12:**
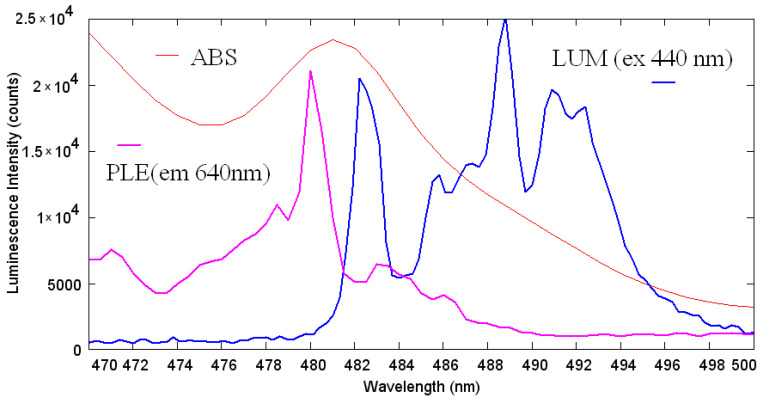
Excitation and emission spectra of Pr_2_(SO_4_)_3_ at 77 K. The absorption spectrum at room temperature is shown for comparison.

Under the excitation at 450 nm, Pr_2_(SO_4_)_3_ exhibits modest luminescence in the red well seen through the filter, with the intensity typical of concentrated rare-earth-containing nonabsorbing materials. The photoluminescence emission spectra excited at the ^3^P_2_ ← ^3^H_6_ transition at 440 nm recorded at room temperature (blue line) and at 77 K (red line), are presented in Figure 11. Both emission spectra are dominated by the ^3^P_0_ → ^3^F_2_ transition with a maximum at 640 nm. The Pr^3+^ ion luminescence in the visible spectral range is expected to include the contributions mainly from ^3^P_0_ and ^1^D_2_ excited states, since ^3^P_2_, commonly, nonradiatively relaxes to ^3^P_0_, and in the hosts with a large phonon cutoff frequency, a considerable probability of nonradiative population of ^1^D_2_ level is assumed. Despite only two luminescent energetic states, the emission spectra of the Pr^3+^ ion are featured by overlapping bands terminating at different low-lying excited states. Another feature is the variability of the intensity distribution over the luminescence bands via the change of crystal field acting onto the Pr^3+^ ion in different hosts, or more specifically, by the change of oscillator strengths and energies of both starting and terminating levels. For example, either ^1^D_2_ → ^3^H_4_ or ^3^P_0_ → ^3^H_6_ or ^3^P_0_ → ^3^F_2_ could be a maximal in different hosts under the excitation via ^3^P_J_. Therefore, the assignment of the Pr^3+^ luminescence bands must be made very carefully in contrast to Eu^3+^, for instance. Figure 12 shows the 77 K excitation spectrum of Pr_2_(SO_4_)_3_ monitored at 640 nm (magenta) and the neighboring part of emission spectrum (blue). Peak at 480 nm (20,833 cm^−1^) must be associated with the zero phonon line (ZPL) of the ^3^P_0_ ← ^3^H_4_ transition, and the weak sideband at longer wavelengths then must be a contribution of thermally distributed phonons corresponding to 77 K. The corresponding peak in the emission spectrum is shifted to longer wavelengths by the ZPL width that can be explained by the reabsorption of emitted radiation within the ZPL width. The longer-wavelength spectral structure in the emission spectrum (in the range 484–500 nm) then must be assigned to the phonon sideband that, in contrast to the excitation process, must not obey the thermal distribution of phonons and is limited by the vibrational spectrum of the local environment of praseodymium ion. Peculiarly, the reabsorption effect for the spectral distribution of luminescence in Pr_2_(SO_4_)_3_ is weaker than that in another self-activated crystal PrAlGe_2_O_7_ [93], where the disappearance of ^3^P_0_ → ^3^H_4_ and ^1^D_2_ → ^3^H_4_ spectral features, with respect to the Pr-doped LaAlGe_2_O_7_ crystal, was observed. One more peculiarity is the absence of the ^3^P_0_ → ^3^F_3_ line that is completely unobservable at the background of the ^3^P_1_ → ^3^F_4_ band. This latter effect cannot be related to the reabsorption; however, it must be associated with a certain dependence of the intensity of this line on the crystal field, like it has been recently observed in [94].

After determining the ^3^P_0_ ZPL position, the assignment of most bands shown in Figure 11 is rather straightforward and becomes consistent with the consideration by Srivastava [95]. Both room temperature and 77K emission spectra are dominated by the luminescence at the ^3^P_0_ → ^3^F_2_ transition peaking at 640 nm. The spectral region in the vicinity of 600 nm contains contributions from two possible channels, namely, from ^3^P_0_ → ^3^H_6_ and ^1^D_2_ → ^3^H_4_. The band peaking at 525 nm is very weak at 77 K and gains more intensity at room temperature; therefore, it must be ascribed to the emission from the thermally populated ^3^P_1_ level to ^3^H_5_. The same behavior reveals the origin of the bands at 675 and 700 nm that are the emissive transitions ^3^P_1_ → ^3^F_3,4_.

## 4. Conclusions

In the present study, the structural and spectroscopic properties, and the thermal stability of Pr_2_(SO_4_)_3_ have been explored for the first time. Anhydrous Pr_2_(SO_4_)_3_ was synthesized by chemical precipitation in hard acids. It was found that Pr_2_(SO_4_)_3_ is hydroscopic at room temperature, leading to the formation of octahydrate Pr_2_(SO_4_)_3_·8H_2_O. Pr_2_(SO_4_)_3_ crystallizes in the monoclinic structure with space group *C*2/*c*, which is typical of sulfates and molybdates of the cerium subgroup. The compound Pr_2_(SO_4_)_3_·8H_2_O is decomposed at temperatures 25–1400 °C in the argon atmosphere and does not undergo pyrohydrolysis or oxidation. The final decomposition product of Pr_2_(SO_4_)_3_·8H_2_O is the intermediate oxide Pr_6_O_11_ being characteristic for the decomposition of oxygen-containing praseodymium compounds. The comparison of the emission spectra recorded at room temperature and at 77 K allowed determining the ZPL position of Pr^3+^ in Pr_2_(SO_4_)_3_ at the ^3^P_0_ → ^3^H_6_ transition and the accurate assignment of the rest of luminescent bands.

## Figures and Tables

**Table 1:** Main parameters of processing and refinement of the Pr_2_(SO_4_)_3_ sample.

Compound	Pr_2_(SO_4_)_3_
Space group	*C*2/*c*
*a*, Å	21.6052 (4)
*b*, Å	6.7237 (1)
*c*, Å	6.9777 (1)
*β*, °	107.9148 (7)
*V*, Å^3^	964.48 (3)
*Z*	4
*2θ*-interval, °	7.5–140
*T*_meas_.	150 °C
Number of reflections	922
Number of refined parameters	71
*R_wp_*, %	2.75
*R_p_*, %	2.16
*R_exp_*, %	2.10
*χ* ^2^	1.31
*R_B_*, %	0.69

**Table 2:** Main parameters of processing and refinement of the Pr_2_(SO_4_)_3_·8H_2_O sample.

Compound	Pr_2_(SO_4_)_3_·8H_2_O
Space group	*C*2/*c*
*a*, Å	13.7058 (2)
*b*, Å	6.8664 (1)
*c*, Å	18.4702 (3)
*β*, °	102.816 (1)
*V*, Å^3^	1694.91 (5)
*Z*	4
*2θ*-interval, °	7–144
*T*_meas_.	24 °C
Number of reflections	1689
Number of refined parameters	48
*R_wp_*, %	6.63
*R_p_*, %	5.08
*R_exp_*, %	2.95
*χ* ^2^	2.24
*R_B_*, %	3.65

**Table 3:** Correlation scheme for the SO_4_^2−^ ion placed into the *C*_1_ and *C*_2_ symmetry positions of the unit cell having *C*_2*h*_ symmetry.

Wavenumber, cm^−1^ [81]	*T_d_* Free Molecule Symmetry	*C*_1_ Site Symmetry	*C*_2*h*_ Unit Cell Symmetry
983	*A*_1_ (ν_1_)	*A*	*A_g_* + *A_u_* + *B_g_* + *B_u_*
450	*E* (ν_2_)	2*A*	2*A_g_* + 2*A_u_* + 2*B_g_* + 2*B_u_*
1105	*F*_2_ (ν_3_)	3*A*	3*A_g_* + 3*A_u_* + 3*B_g_* + 3*B_u_*
611	*F*_2_ (ν_4_)	3*A*	3*A_g_* + 3*A_u_* + 3*B_g_* + 3*B_u_*
	*T_d_* Free molecule symmetry	*C*_2_ Site symmetry	*C*_2*h*_ Unit cell symmetry
983	*A*_1_ (ν_1_)	*A*	*A_g_* + *A_u_*
450	*E* (ν_2_)	2*A*	2*A_g_* + 2*A_u_*
1105	*F*_2_ (ν_3_)	*A* + 2*B*	*A_g_* + *A_u_* + 2*B_g_* + 2*B_u_*
611	*F*_2_ (ν_4_)	*A* + 2*B*	*A_g_* + *A_u_* + 2*B_g_* + 2*B_u_*

**Table 4:** Standard enthalpies of praseodymium sulfate formation.

Compound	∆*H*°*f*, kJ/mol
Pr_2_(SO_4_)_3_·8H_2_O	−5361.2
Pr_2_(SO_4_)_3_	−3317.9
Pr_2_O_2_SO_4_	−2224.3

**Table 5:** Kinetic parameters of the decomposition of praseodymium sulfates.

Number of Reaction	Chemical Equation	A	E_a_, kJ/mol
1	Pr_2_(SO_4_)_3_·8H_2_O → Pr_2_(SO_4_)_3_ + 8H_2_O	6 × 10^6^	77
2	Pr_2_(SO_4_)_3_ → Pr_2_O_2_SO_4_ + 2SO_2_ + O_2_	1 × 10^10^	303
3	6Pr_2_O_2_SO_4_ → 2Pr_6_O_11_ + 6SO_2_ + O_2_	2 × 10^8^	323

## Supplementary Materials

The following supporting information can be downloaded at: https://www.mdpi.com/article/10.3390/molecules27133966/s1, Figure S1: The digital image of (a) Pr_2_(SO_4_)_3_ and (b) Pr_2_(SO_4_)_3_·8H_2_O powder under the Sun day illumination; Figure S2: Four XRD patterns measured for the Pr_2_(SO_4_)_3_ sample with 30 min intervals on keeping in the laboratory air at ambient conditions; Figure S3: Difference Rietveld plots of Pr_2_(SO_4_)_3_ at different temperatures: (a) T = 30 °C; (b) T = 60 °C; (c) T = 90 °C; (d) T = 120 °C; (e) T = 150 °C; (f) T = 180 °C; (g) T = 210 °C; (h) T = 240 °C; (i) T = 270 °C; Figure S4: Brillouin zone of Pr_2_(SO_4_)_3_; Figure S5: Raman spectra for Pr_2_(SO_4_)_3_ recorded at 1064 and 532.1 nm excitation wavelengths; Figure S6: Comparison of the high-frequency part of Raman spectra for Eu_2_(SO_4_)_3_ and Pr_2_(SO_4_)_3_; Figure S7: Decomposition of the high-frequency part of Pr_2_(SO_4_)_3_ Raman spectra; Figure S8: Decomposition of Raman spectra of Pr_2_(SO_4_)_3_ in the range of ν_4_ vibrations; Figure S9: Heat effect showing up in dependence of heating rate for processes: (a) Pr_2_(SO_4_)_3_8H_2_O → Pr_2_(SO_4_)_3_ + 8H_2_O; (b) Pr_2_(SO_4_)_3_ → Pr_2_O_2_SO_4_ + 2SO_2_ + O_2_; (c) 6 Pr_2_O_2_SO_4_→ 2Pr_6_O_11_ + 6SO_2_ + O_2_(heating rate: I-3 °C/min, II-5 °C/min, III-10 °C/min, IV-15 °C/min); Figure S10: Linearity in the manifestation of the maxima of thermal effects depending on the heating rate Table S1: Fractional atomic coordinates and isotropic displacement parameters (Å^2^) in Pr_2_(SO_4_)_3_; Table S2: Main bond lengths (Å) in Pr_2_(SO_4_)_3_; Table S3: Main parameters of processing and refinement of the Pr_2_(SO_4_)_3_ sample at T = 30–270 °C; Table S4: Fractional atomic coordinates (Å) and occupancies of Pr_2_(SO_4_)_3_·8H_2_O; Table S5: Main bond lengths (Å) in Pr_2_(SO_4_)_3_·8H_2_O.
