# Supplementary material for: Exploration of the Crystal Structure and Thermal and Spectroscopic Properties of Monoclinic Praseodymium Sulfate Pr2(SO4)3
Source: Molecules. 2022 Jun 21;27(13):3966. doi: 10.3390/molecules27133966 (PMC9267875; doi:10.3390/molecules27133966)
Supplement: Supplementary file 1 [file molecules-27-03966-s001.zip › Pr2(SO4)3_Support_20.06.22.pdf]

Supporting materials

Exploration of the Crystal Structure and Thermal and Spectroscopic Properties of Monoclinic Praseodymium Sulfate $Pr_2(SO_4)_3$

Yuriy G. Denisenko[1,2,3], Victor V. Atuchin[4,5,6,7,8,*], Maxim S. Molokeev[9,10,11], Alexander E. Sedykh[3], Nikolay A. Khritokhin[1], Aleksandr S. Aleksandrovsky[12,13], Aleksandr S. Oreshonkov[14,15], Nikolai P. Shestakov[14], Sergey V. Adichtchev[16], Alexey M. Pugachev[16], Elena I. Sal'nikova[1,17], Oleg V. Andreev[1], Illaria A. Razumkova[1] and Klaus Müller-Buschbaum[3,18]

[1]Department of Inorganic and Physical Chemistry, Tyumen State University, 625003 Tyumen, Russia; yu.g.denisenko@gmail.com (Y.G.D.); kna@utmn.ru (N.A.K.); elenasalnikova213@gmail.com (E.I.S.); o.v.andreev@utmn.ru (O.V.A.); razumkova@list.ru (I.A.R.)

[2]Department of General and Special Chemistry, Industrial University of Tyumen, 625000 Tyumen, Russia

[3]Institute of Inorganic and Analytical Chemistry, Justus-Liebig-University Giessen, 35392 Giessen, Germany; al-exander.sedykh@anorg.chemie.uni-giessen.de (A.E.S.); klaus.mueller-buschbaum@anorg.chemie.uni-giessen.de (K.M.-B.)

[4]Laboratory of Optical Materials and Structures, Institute of Semiconductor Physics, SB RAS, 630090 Novosibirsk, Russia

[5]Research and Development Department, Kemerovo State University, 650000 Kemerovo, Russia

[6]Department of Applied Physics, Novosibirsk State University, 630090 Novosibirsk, Russia

[7]Department of Industrial Machinery Design, Novosibirsk State Technical University, 630073 Novosibirsk, Russia

[8]R&D Center "Advanced Electronic Technologies", Tomsk State University, Tomsk 634034, Russia

[9]Laboratory of Crystal Physics, Kirensky Institute of Physics, Federal Research Center KSC SB RAS, 660036 Krasnoyarsk, Russia; msmolokeev@mail.ru

[10]School of Engineering Physics and Radio Electronics, Siberian Federal University, 660041 Krasnoyarsk, Russia

[11]Department of Physics, Far Eastern State Transport University, 680021 Khabarovsk, Russia

[12]Laboratory of Coherent Optics, Kirensky Institute of Physics Federal Research Center KSC SB RAS, 660036 Krasnoyarsk, Russia; aleksandrovsky@kirensky.ru

[13]Institute of Nanotechnology, Spectroscopy and Quantum Chemistry, Siberian Federal University, 660041 Krasnoyarsk, Russia

[14]Laboratory of Molecular Spectroscopy, Kirensky Institute of Physics Federal Research Center KSC SB RAS, 660036 Krasnoyarsk, Russia; oreshonkov@iph.krasn.ru (A.S.O.); nico@iph.krasn.ru (N.P.S.)

[15]School of Engineering and Construction, Siberian Federal University, 660041 Krasnoyarsk, Russia

[16]Institute of Automation and Electrometry, Russian Academy of Sciences, 630090 Novosibirsk, Russia; adish2@ngs.ru (S.V.A.); apg@iae.nsk.su (A.M.P.)

[17]Research Department, Northern Trans-Ural Agricultural University, 625003 Tyumen, Russia

[18]Center for Materials Research (LaMa), Justus-Liebig-University Giessen, 35392 Giessen, Germany

*Correspondence: atuchin@isp.nsc.ru

Table S1. Fractional atomic coordinates and isotropic displacement parameters (Å^2) in $Pr_2(SO_4)_3$

	x	*y*	*z*	B_{iso}
Pr	0.13171 (3)	0.65109 (12)	0.12749 (11)	0.74 (5)
S1	0.18297 (15)	0.1864 (4)	0.1763 (5)	0.60 (8)
S2	0	0.6621 (8)	1/4	1.10 (11)
O1	0.1471 (3)	-0.0031 (8)	0.1270 (11)	0.51 (8)
O2	0.1513 (3)	0.3167 (11)	0.3022 (10)	0.51 (8)
O3	0.2523 (3)	0.1488 (11)	0.2861 (8)	0.51 (8)
O4	0.1755 (3)	0.3038 (12)	-0.0083 (9)	0.51 (8)
O5	0.0554 (3)	0.5410 (10)	0.3544 (9)	0.51 (8)
O6	0.0229 (3)	0.7783 (9)	0.1120 (8)	0.51 (8)

Table S2. Main bond lengths (Å) in $Pr_2(SO_4)_3$

Pr—O1^{i}	2.349 (6)	S1—O1	1.476 (6)
Pr—O2	2.530 (7)	S1—O2	1.542 (7)
Pr—O2^{ii}	2.443 (6)	S1—O3	1.479 (5)
Pr—O3^{iii}	2.391 (4)	S1—O4	1.477 (7)
Pr—O4^{iv}	2.443 (6)	S2—O5	1.446 (6)
Pr—O5^{ii}	2.466 (6)	S2—O6	1.441 (6)
Pr—O6	2.473 (4)		

Symmetry codes for:(i) *x*, *y*+1, *z*; (ii) *x*, -*y*+1, *z*-1/2; (iii) -*x*+1/2, *y*+1/2, -*z*+1/2; (iv) *x*, -*y*+1, *z*+1/2; (v) -*x*, *y*, -*z*+1/2.

Table S3. Main parameters of processing and refinement of the $Pr_2(SO_4)_3$ sample at T = 30-270 ºC

T, ºC	Space group	Cell parameters (º, Å), Cell volume (Å^3)	R_{wp}, R_p(%), χ^2
30	*C*2/*c*	a = 21.5713 (7), b = 6.7161 (2), c = 6.9634 (4), β = 107.687 (2), V = 961.13 (5)	5.83, 4.49, 1.08
60	*C*2/*c*	a = 21.5724 (8), b = 6.7161 (3), c = 6.9645 (3), β = 107.698 (2), V = 961.28 (7)	5.78, 4.49, 1.07
90	*C*2/*c*	a = 21.5773 (9), b = 6.7168 (3), c = 6.9660 (3), β = 107.712 (2), V = 961.74 (7)	5.77, 4.48, 1.07
120	*C*2/*c*	a = 21.5815 (7), b = 6.7179 (2), c = 6.9681 (2), β = 107.738 (2), V = 962.22 (5)	5.81, 4.57, 1.08
150	*C*2/*c*	a = 21.5866 (9), b = 6.7193 (3), c = 6.9700 (3), β = 107.778 (2), V = 962.69 (7)	5.96, 4.62, 1.11
180	*C*2/*c*	a = 21.5985 (6), b = 6.7215 (2), c = 6.9764 (2), β = 107.803 (2), V = 964.29 (5)	5.81, 4.51, 1.06
210	*C*2/*c*	a = 21.6057 (9), b = 6.7222 (3), c = 6.9820 (3), β = 107.870 (2), V = 965.12 (7)	5.89, 4.57, 1.08
240	*C*2/*c*	a = 21.6134 (7), b = 6.7242 (2), c = 6.9853 (2), β = 107.906 (2), V = 966.02 (6)	5.89, 4.47, 1.08
270	*C*2/*c*	a = 21.6193 (9), b = 6.7256 (3), c = 6.9888 (3), β = 107.935 (2), V = 966.80 (8)	5.89, 4.57, 1.08

Table S4. Fractional atomic coordinates (Å) and occupancies of $Pr_2(SO_4)_3{\cdot}8H_2O$

Atom	*x*	*y*	*z*	Occ.
Pr1	0.16914 (14)	0.4763 (3)	0.39378 (13)	1
O1	0.3501 (12)	0.512 (3)	0.4574 (9)	1
O2	0.0155 (16)	0.664 (3)	0.3590 (13)	1
O3	0.0386 (15)	0.261 (3)	0.4350 (10)	1
O4	0.2665 (10)	0.507 (4)	0.2943 (8)	1
S1	0.2119 (6)	0.5258 (16)	0.5884 (5)	1
O5	0.1984 (14)	0.821 (3)	0.3976 (13)	1
O6	0.1600 (13)	0.521 (4)	0.6445 (11)	1
O7	0.1487 (13)	0.593 (3)	0.5226 (11)	1
O8	0.2497 (14)	0.169 (3)	0.4250 (12)	1
S2	-0.0073 (14)	0.1809 (11)	0.2554 (11)	1
O9	0.0849 (16)	0.315 (3)	0.2924 (12)	1
O10	-0.0370 (11)	0.037 (3)	0.3116 (9)	1
H1	0.3814	0.4312	0.4838	1
H2	0.361	0.6166	0.4845	1
H3	-0.0434	0.6132	0.3549	1
H4	0.0076	0.7843	0.3477	1
H5	0.0064	0.1908	0.4028	1
H6	-0.0066	0.3184	0.4527	1
H7	0.2369	0.4741	0.2524	1
H8	0.3197	0.5429	0.3007	1

Table S5. Main bond lengths (Å) in $Pr_2(SO_4)_3{\cdot}8H_2O$

Pr1—O1	2.509 (13)	Pr1—O9	2.26 (2)
Pr1—O2	2.430 (17)	S1—O5^{ii}	1.594 (18)
Pr1—O3	2.561 (17)	S1—O6	1.382 (18)
Pr1—O4	2.508 (13)	S1—O7	1.40 (2)
Pr1—O5	2.398 (19)	S1—O8^{i}	1.48 (2)
Pr1—O7	2.583 (19)	S2—O9^{iii}	1.554 (19)
Pr1—O8^{i}	3.43 (2)	S2—O10^{iii}	1.669 (18)
Pr1—O8	2.392 (19)		

Symmetry codes: (i) -*x*+1/2, -*y*+1/2, -*z*+1; (ii) -*x*+1/2, -*y*+3/2, -*z*+1; (iii) -*x*, *y*, -*z*+1/2

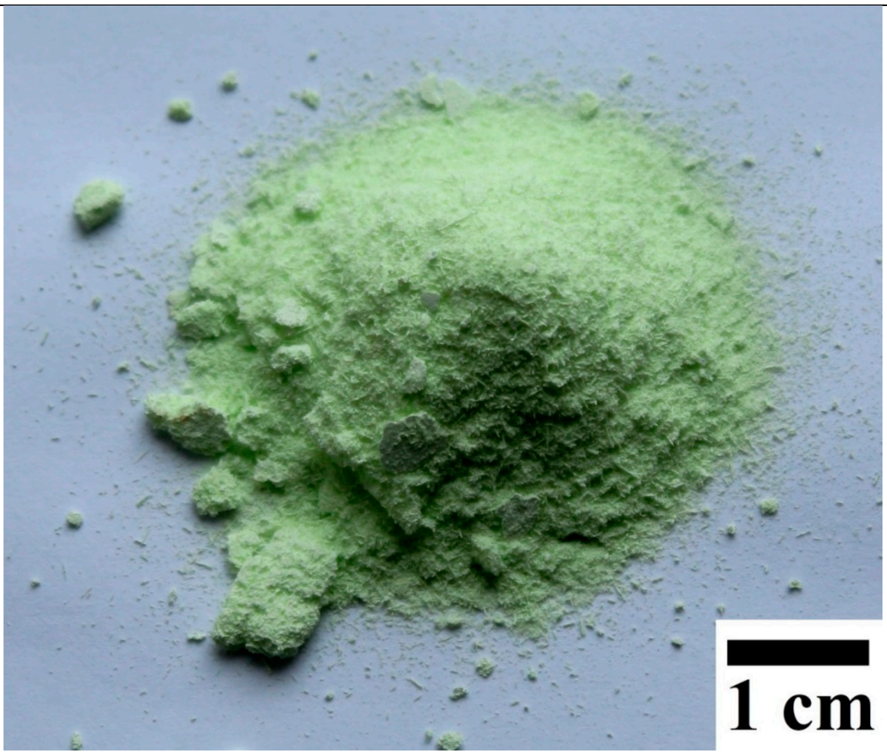

a

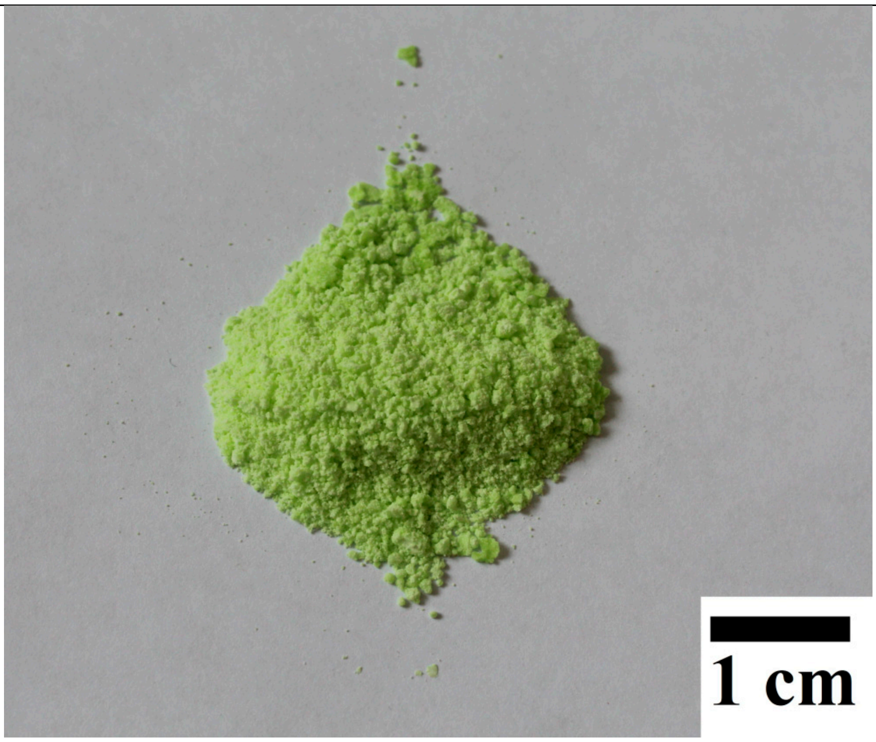

b

Figure S1. The digital image of (a) $Pr_2(SO_4)_3$ and (b) $Pr_2(SO_4)_3 \cdot 8H_2O$ powder under the Sun day illumination.

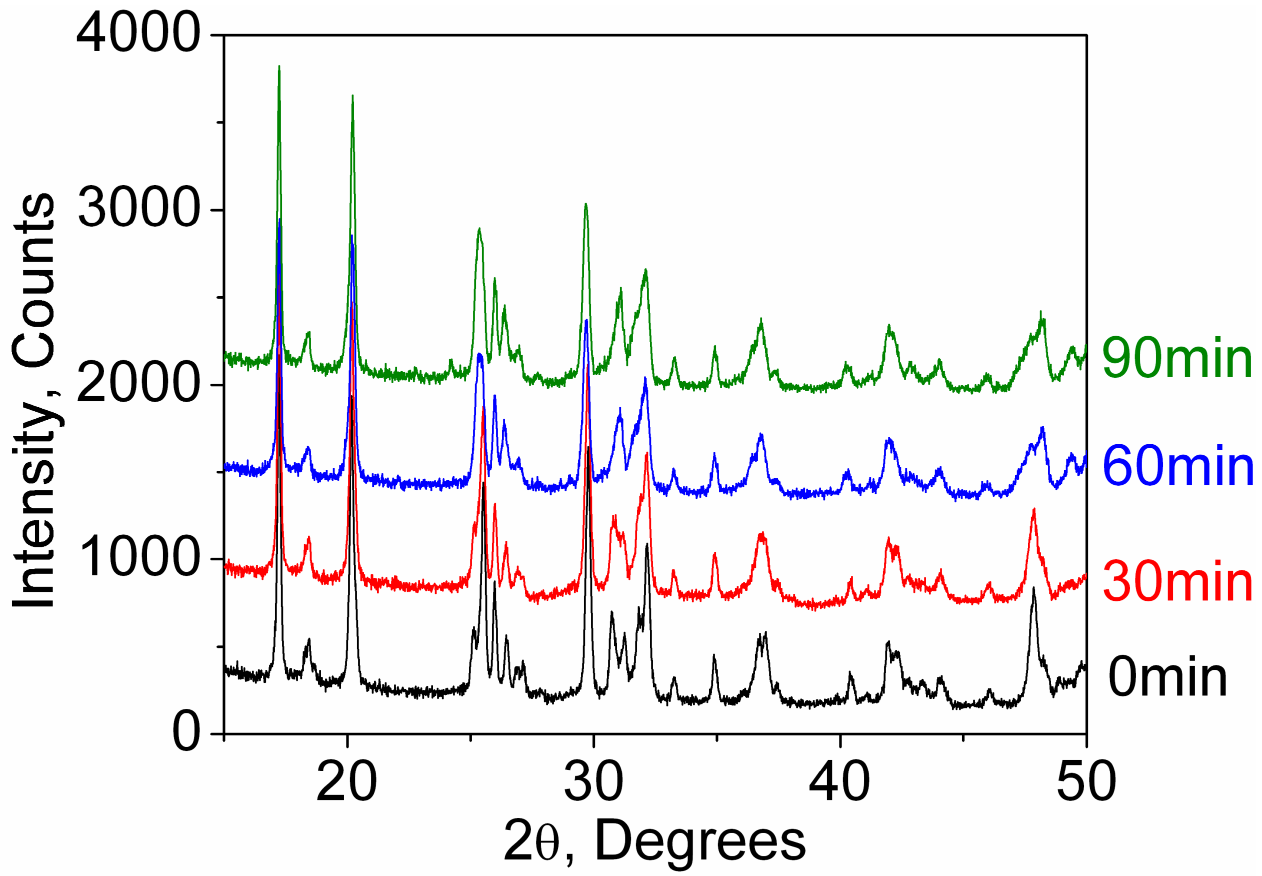

Figure S2. Four XRD patterns measured for the $Pr_2(SO_4)_3$ sample with 30 min intervals on keeping in the laboratory air at ambient conditions.

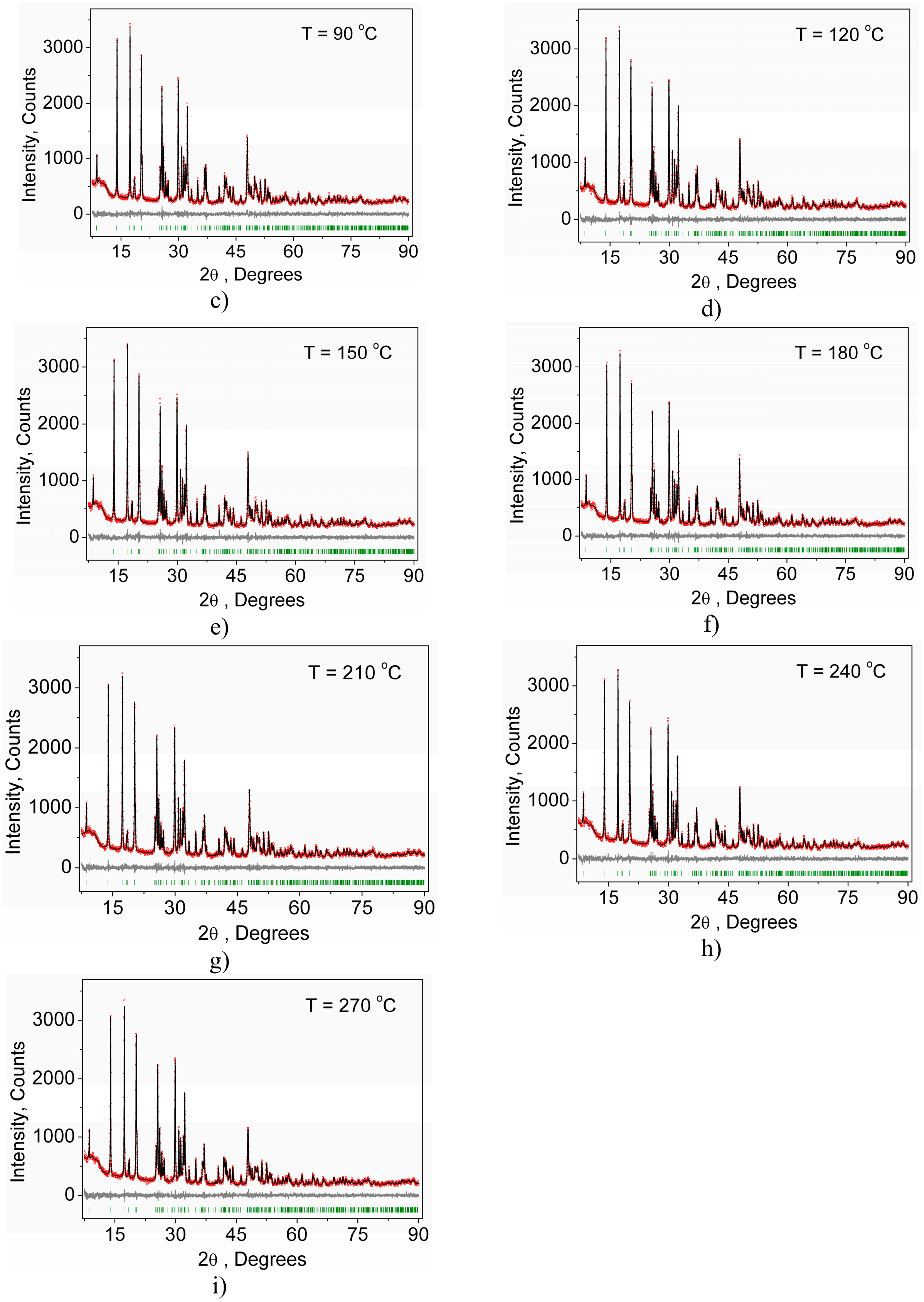

Figure S3. Difference Rietveld plots of $Pr_2(SO_4)_3$ at different temperatures: a) T = 30 ºC; b) T = 60 ºC; c) T = 90 ºC; d) T = 120 ºC; e) T = 150 ºC; f) T = 180 ºC; g) T = 210 ºC; h) T = 240 ºC; i) T = 270 ºC.

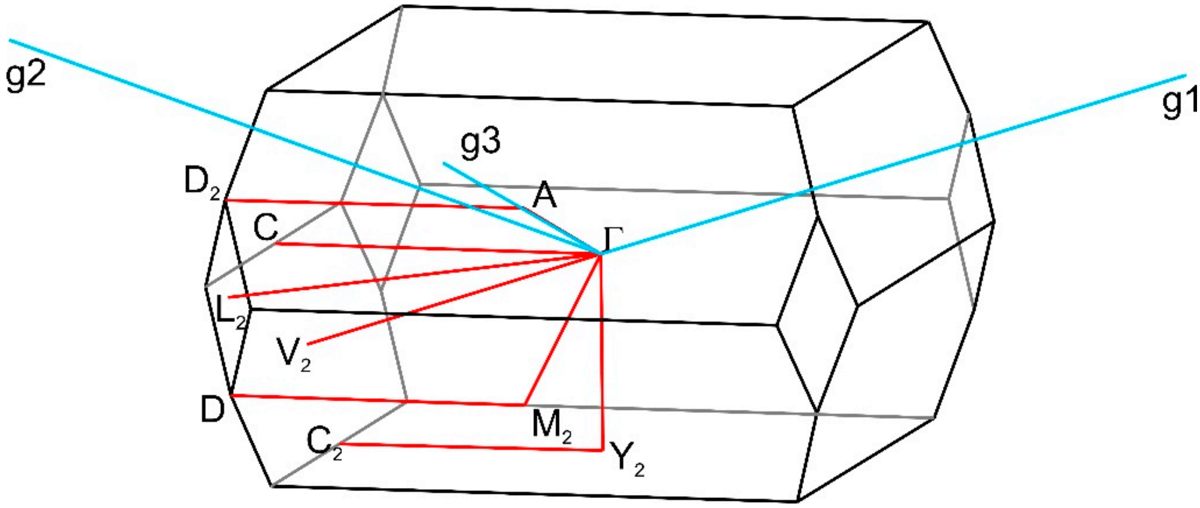

Figure S4. Brillouin zone of $Pr_2(SO_4)_3$.

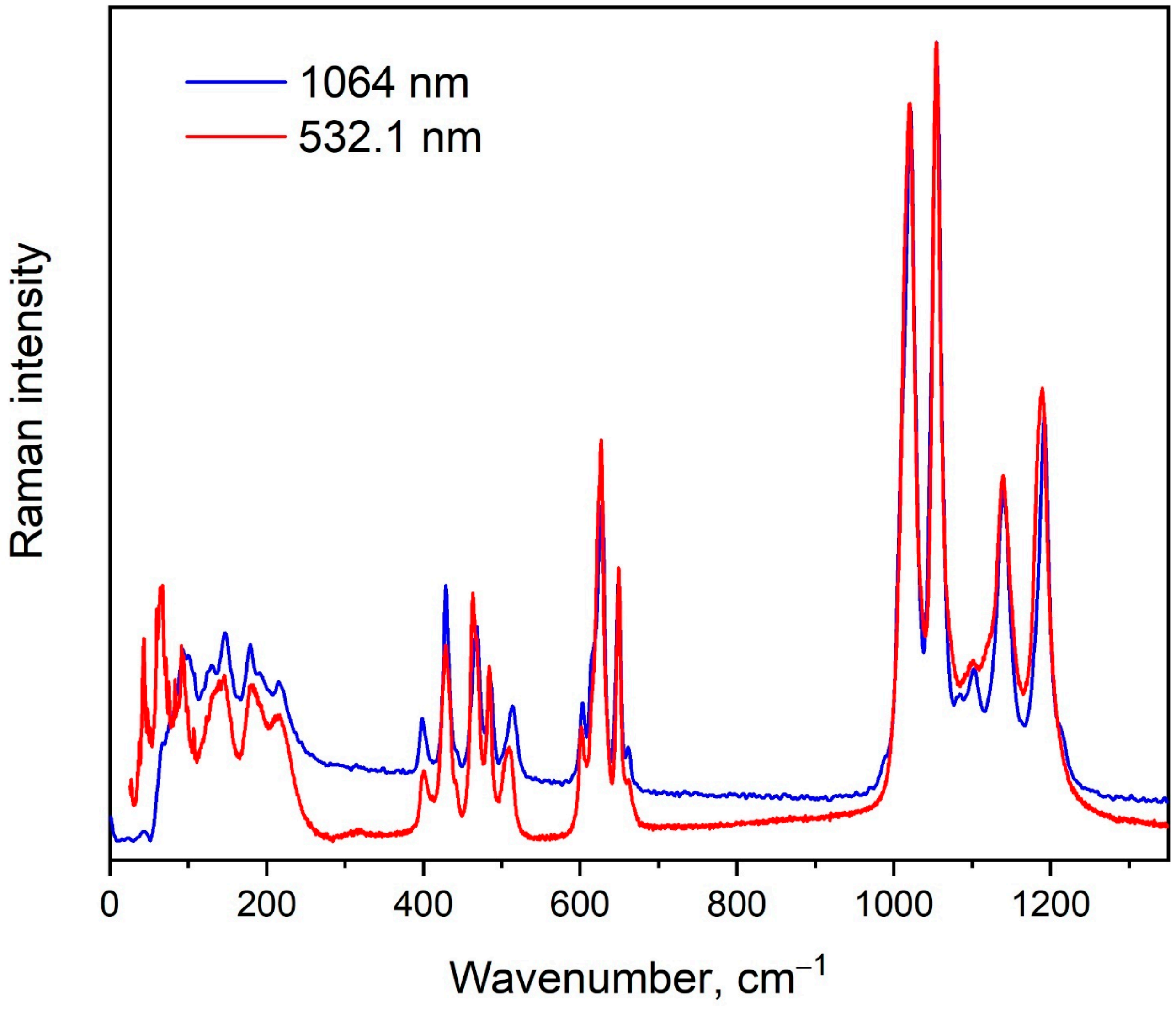

Figure S5. Raman spectra for $Pr_2(SO_4)_3$ recorded at 1064 and 532.1 nm excitation wavelengths.

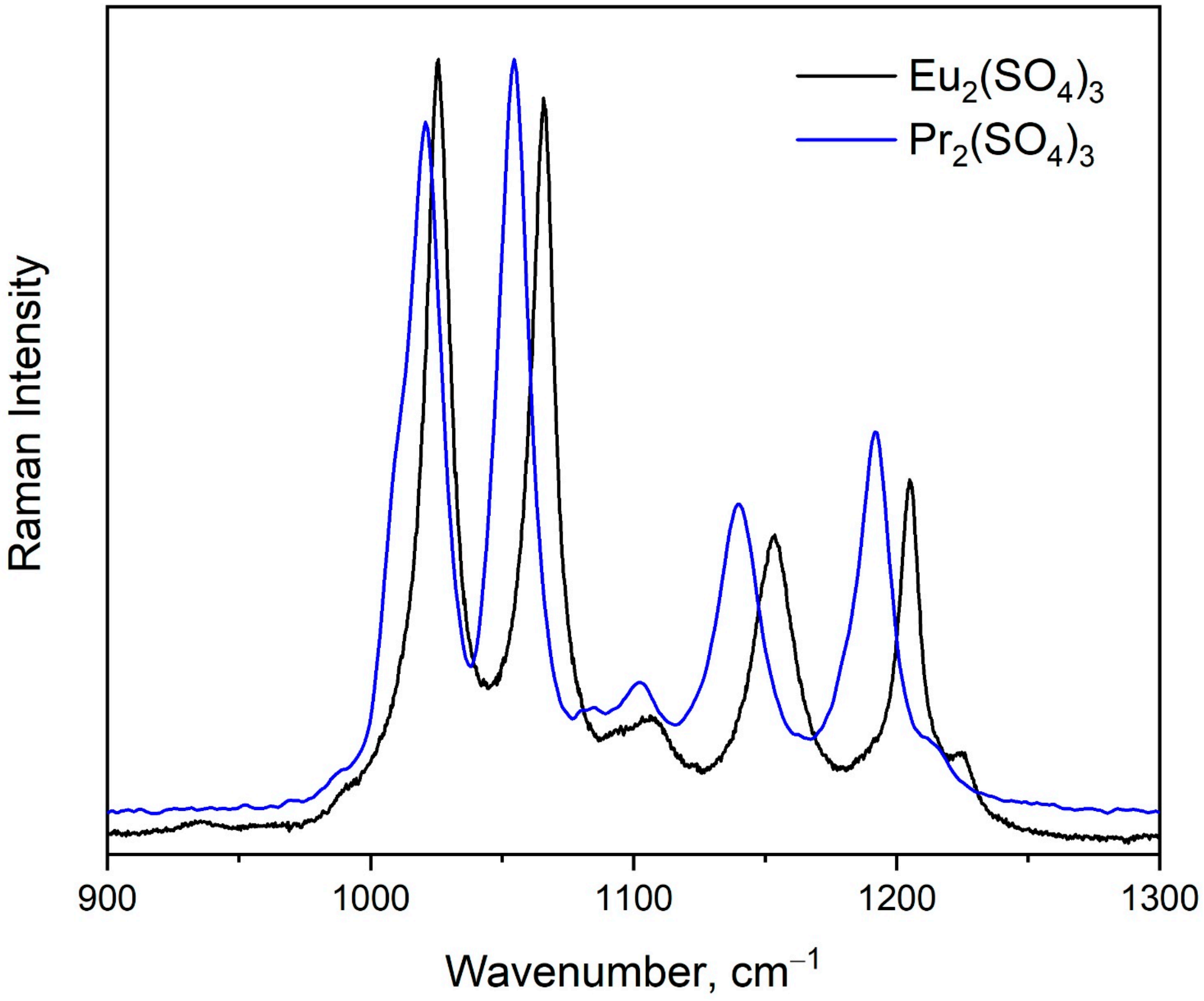

Figure S6. Comparison of the high-frequency part of Raman spectra for $Eu_2(SO_4)_3$ and $Pr_2(SO_4)_3$.

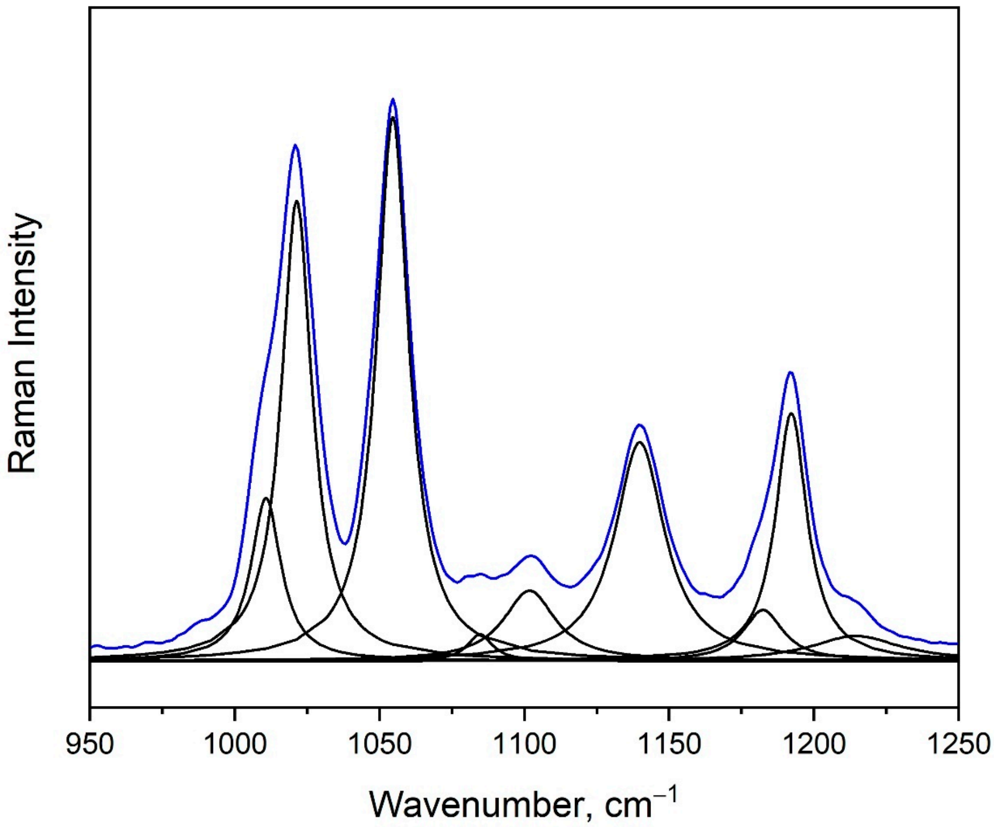

Figure S7. Decomposition of the high-frequency part of $Pr_2(SO_4)_3$ Raman spectra.

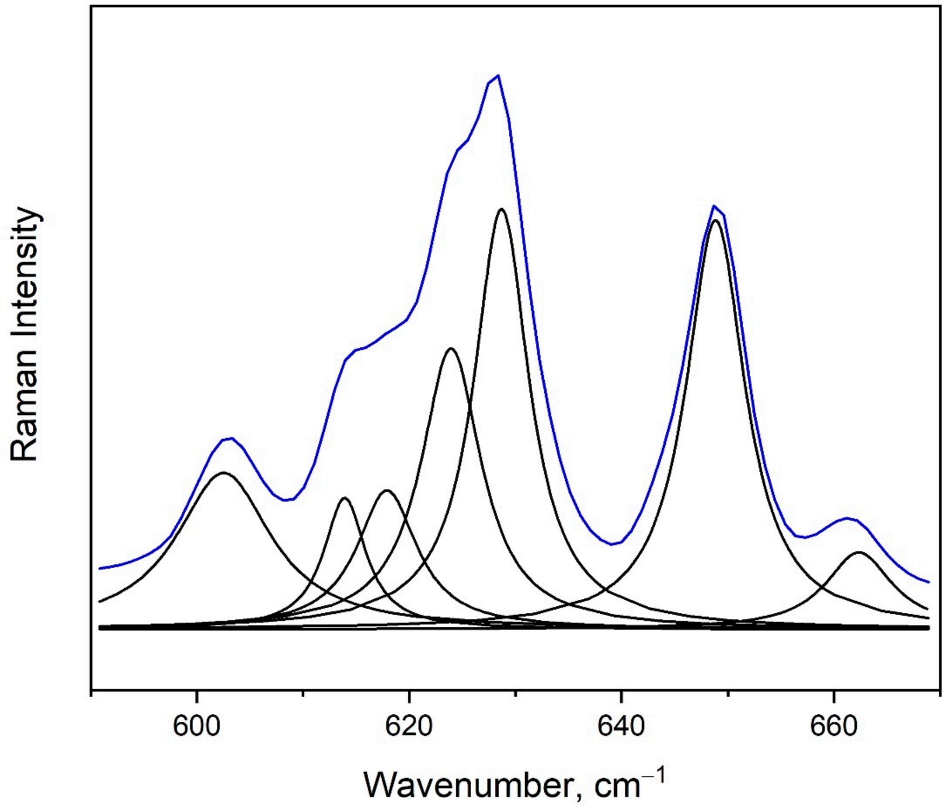

Figure S8. Decomposition of Raman spectra of $Pr_2(SO_4)_3$ in the range of ν_4 vibrations.

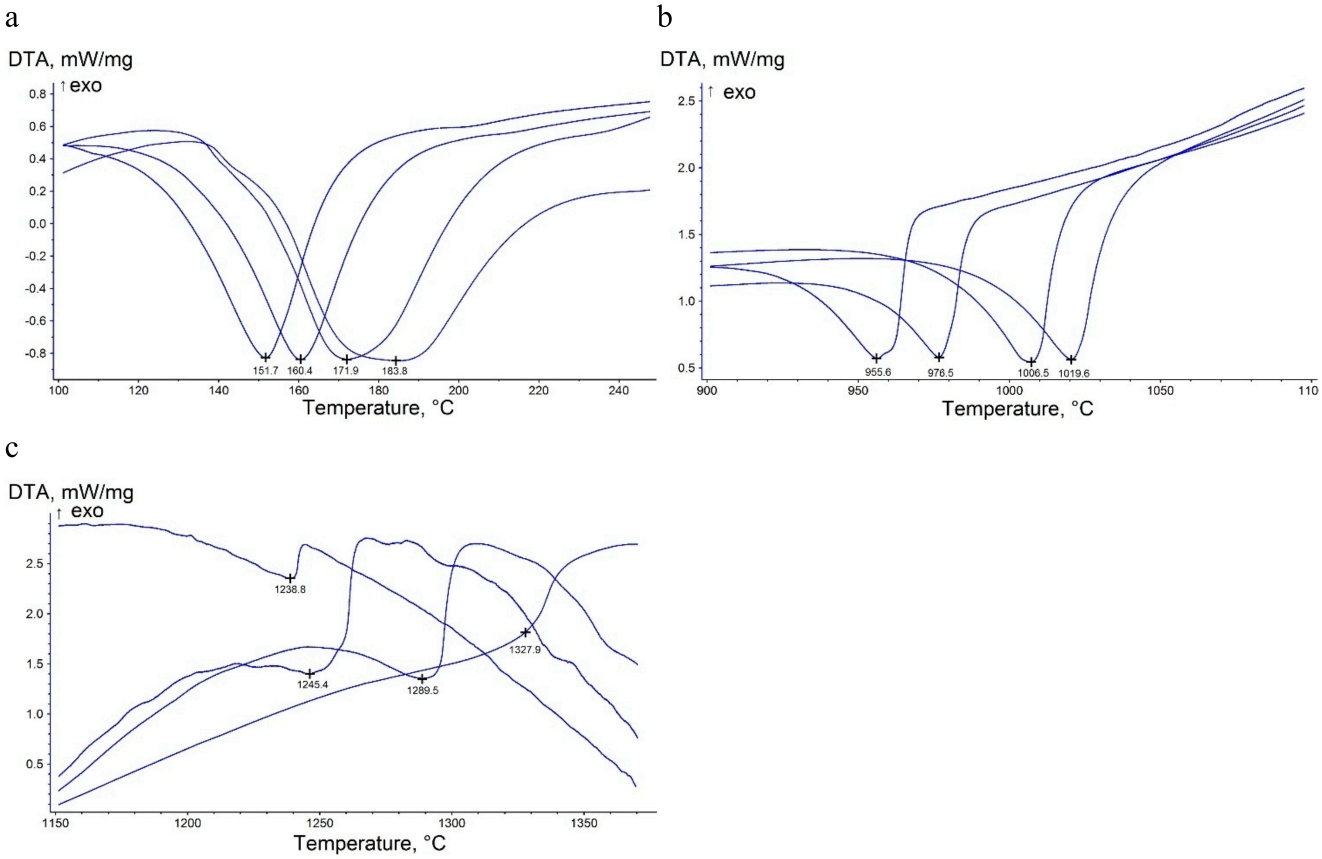

Figure S9. Heat effect showing up in dependence of heating rate for processes: (a) $Pr_2(SO_4)_3 \cdot 8H_2O \rightarrow Pr_2(SO_4)_3 + 8H_2O$; (b) $Pr_2(SO_4)_3 \rightarrow Pr_2O_2SO_4 + 2SO_2 + O_2$; (c) $6\ Pr_2O_2SO_4 \rightarrow 2Pr_6O_{11} + 6SO_2 + O_2$(heating rate: I- 3 ºC/min, II- 5ºC/min, III- 10ºC/min, IV- 15ºC/min).

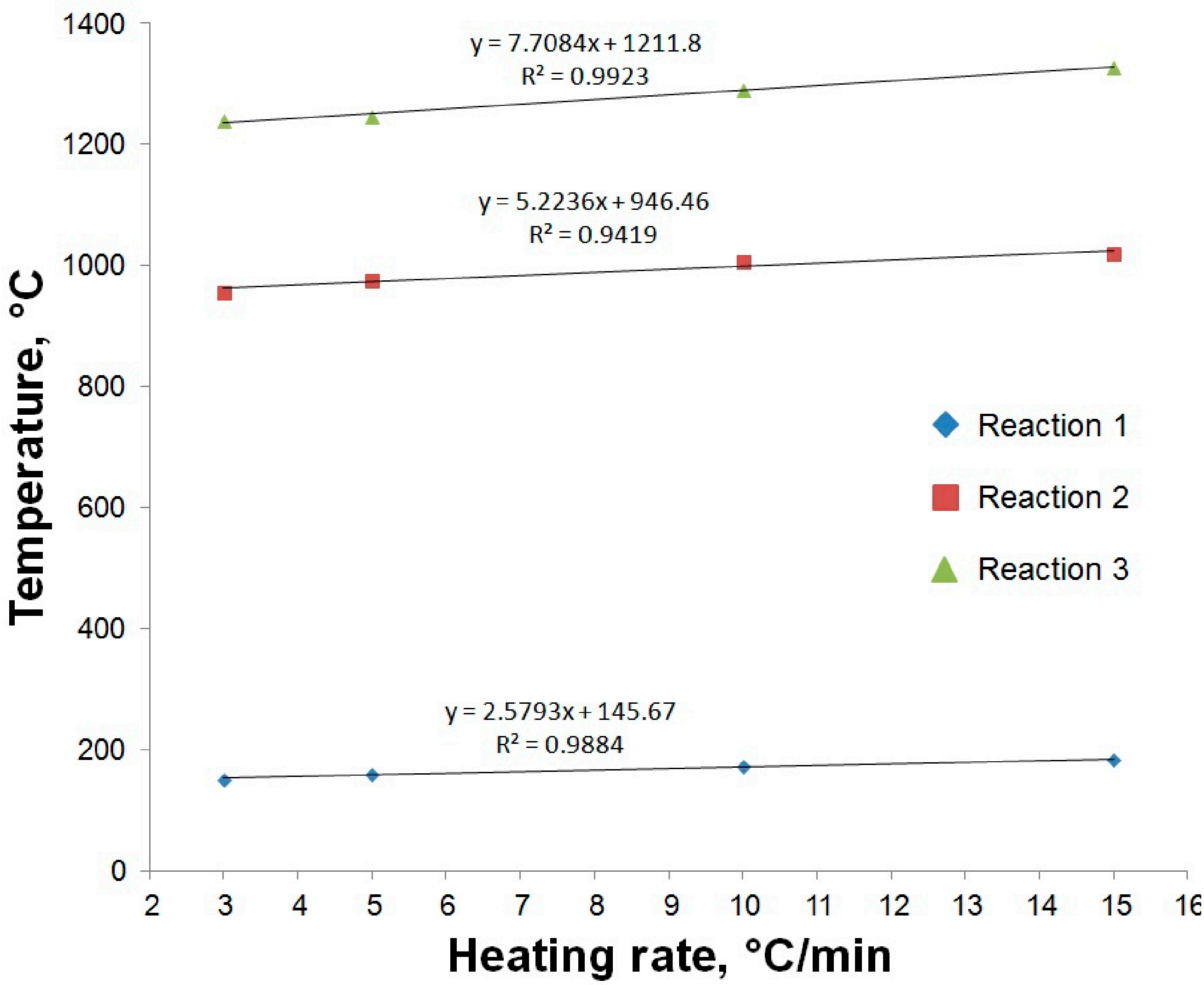

Figure S10. Linearity in the manifestation of the maxima of thermal effects depending on the heating rate.
